# Peroxin FgPEX22-Like Is Involved in FgPEX4 Tethering and *Fusarium graminearum* Pathogenicity

**DOI:** 10.3389/fmicb.2021.756292

**Published:** 2021-12-10

**Authors:** Li Zhang, Chunjie Liu, Mingyu Wang, Yilin Tao, Yuancun Liang, Jinfeng Yu

**Affiliations:** Key Laboratory of Agricultural Microbiology, College of Plant Protection, Shandong Agricultural University, Tai’an, China

**Keywords:** *Fusarium graminearum*, *FgPEX22-like*, *FgPEX4*, pathogenicity, peroxisome

## Abstract

Peroxisomes are essential organelles that play important roles in a variety of biological processes in eukaryotic cells. To understand the synthesis of peroxisomes comprehensively, we identified the gene *FgPEX22-like*, encoding FgPEX22-like, a peroxin, in *Fusarium graminearum*. Our results showed that although FgPEX22-like was notably different from other peroxins (PEX) in *Saccharomyces cerevisiae*, it contained a predicted PEX4-binding site and interacted with FgPEX4 as a rivet protein of FgPEX4. To functionally characterize the roles of *FgPEX22-like* in *F. graminearum*, we performed homologous recombination to construct a deletion mutant (Δ*PEX22-like*). Analysis of the mutant showed that *FgPEX22-like* was essential for sexual and asexual reproduction, fatty acid utilization, pathogenicity, and production of the mycotoxin deoxynivalenol. Deletion of *FgPEX22-like* also led to increased production of lipid droplets and decreased elimination of reactive oxygen species. In addition, FgPEX22-like was required for the biogenesis of Woronin bodies. Taken together, our data demonstrate that FgPEX22-like is a peroxin in *F. graminearum* that interacts with PEX4 by anchoring PEX4 at the peroxisomal membrane and contributes to the peroxisome function in *F. graminearum*.

## Introduction

Fusarium head blight (FHB), caused by *F. graminearum* (teleomorph *Gibberella zeae*), is an acute disease of wheat and barley worldwide. In addition to causing severe crop yield losses, *F. graminearum* produces deoxynivalenol (DON), a mycotoxin that also acts as a virulence factor to facilitate wheat infection, and is a menace to the health of humans and animals (Desjardins et al., 1993; Pestka and Smolinski, 2005; Dean et al., 2012).

Peroxisomes are single-membrane-bound organelles present in most eukaryotic organisms and are relevant to multifarious metabolic conversions. For instance, in eukaryotic cells, peroxisomes are involved in methanol oxidation, disposal of ROS, and utilization of carbon sources (Lazarow and Fujiki, 1985; Wanders, 2004; Gould et al., 2010). In yeasts, peroxisomes are the unique sites of fatty acid β-oxidation (Hiltunen et al., 2003), whereas in plants, they are essential for host resistance, embryo development, synthesis of phytohormones, and the glyoxylate cycle (Hu et al., 2012; Shabab, 2013). In addition, peroxisomes are involved in several physiological processes in mammals, such as the synthesis of cholesterol, plasmalogens, and bile acids (Wanders and Waterham, 2010). Zellweger syndrome (ZS), a prototypic peroxisome biogenesis disorder (PBD) with the most severe phenotype in humans, may result in the absence of functional peroxisomes (Wanders, 2004; Faust et al., 2005).

Proteins related to peroxisomal biogenesis are termed peroxins and are encoded by *PEX* genes. So far, more than 30 *PEX*s have been identified in various organisms (Distel et al., 1996; Pieuchot and Jedd, 2012). As peroxisomes do not contain any genetic material, their peroxisomal membrane proteins (PMPs) and matrix proteins are encoded in the nucleus, synthesized in the cytoplasm, and then imported into the peroxisomes. For example, PEX5 is a cycling receptor for the import of PMPs containing the peroxisomal targeting signal type 1 (PTS1). The PEX5 import cycle involves the following steps: protein containing PTS1 is recognized by PEX5 in the cytosol, the PEX5–cargo complex docks at the peroxisomal membrane, the cargo is translocated into the peroxisomal lumen, and after dissociation PEX5 is recycled back to the cytosol for a new import cycle (Albertini et al., 1997; Wang et al., 2003; Stanley et al., 2006; Platta et al., 2007, 2008; Meinecke et al., 2010). During the last step, PEX5 must be mono-ubiquitinated by the ubiquitin-conjugating enzyme PEX4 and its membrane-anchor PEX22 (Collins et al., 2000; Zolman et al., 2005). We previously showed that PEX4 is indispensable for peroxisome function in *F. graminearum* and its pathogenesis (Zhang et al., 2019b). More attention has been paid to the function of peroxins in fungi that are pathogenic to plants. There are several peroxins in *F. graminearum* involved in the peroxisome life cycle that have been characterized. For instance, FgPEX1, FgPEX2, FgPEX4, FgPEX5, FgPEX6, FgPEX7, FgPEX10, FgPEX12, FgPEX13, FgPEX14, and FgPEX33 are involved in mycotoxin biosynthesis, pathogenicity, and pexophagy (Min et al., 2012; Chen et al., 2018; Zhang et al., 2019a,b; Wang et al., 2020). In *Magnaporthe oryzae*, MoPEX5, MoPEX6, MoPEX7, MoPEX14, MoPEX19, and MoPEX11 family peroxins are involved in matrix protein import and peroxisomal fission processes (Deng et al., 2013; Wang et al., 2013, 2015; Li et al., 2014). Peroxisome studies in other filamentous fungi, such as *Neurospora crassa*, *Colletotrichum orbiculare*, and *Aspergillus nidulans*, are becoming more common (Hynes et al., 2008; Fujihara et al., 2010; Managadze et al., 2010).

In *S. cerevisiae*, PEX22 plays a key role in tethering the PEX4, a ubiquitin-conjugating enzyme, to the peroxisome and is associated with PEX5 receptor recycling (Williams et al., 2012). Previous studies have shown that most of the *PEX* genes was contained in filamentous fungi, but except for *PEX15*, *PEX17*, *PEX18*, *PEX21*, and *PEX22* (Heinemann et al., 2004). Until the functions of FAM1 in *C. orbiculare* were described, functions similar to those of PEX22 was discovered (Kubo et al., 2015). However, whether a similar protein exists in *F. graminearum* and whether it would possess PEX22 function is unclear.

Herein, we identified *FgPEX22-like*, which encodes a peroxin FgPEX22-like, which is the functional ortholog of the PEX22 of *Saccharomyces cerevisiae*. FgPEX22-like was able to interact with FgPEX4 and was essential for the subcellular localization of FgPEX4. Functional analysis provided evidence that *FgPEX22-like* plays important roles in sexual and asexual reproduction, carbon source utilization, pathogenicity, and cell wall integrity. Importantly, FgPEX22-like was essential for the biosynthesis of Woronin bodies.

## Materials and Methods

### Fungal Strains and Growth Conditions

For performing mycelial growth assays, the WT *F. graminearum* strain PH-1, *FgPEX4* deletion mutants, and other transformants generated in this study were grown on potato dextrose agar (PDA) medium in a 25°C incubator. For the aerial hyphal growth assay, all strains were inoculated into test tubes (1.5 cm diameter) containing 5 mL PDA medium and grown at 25°C for 5 days. To study the integrity of cell membranes and cell walls, all strains were grown as previously reported on CM medium supplemented with 0.01% SDS as a cell membrane-damaging agent and 0.2% Congo red as a cell wall-damaging agent (Gavric et al., 2007; Chayakulkeeree et al., 2008; Qin et al., 2015).

### Yeast Two-Hybrid Assay and Co-immunoprecipitation

The Y2H assay was performed using the Matchmaker GAL4 Two-Hybrid System 3 (Clontech) according to the manufacturer’s instructions. Full-length complementary DNA (cDNA) of *FgPEX22-like* and *FgPEX4* was PCR amplified using the primer pairs P22-AD-F/22R and P4-AD-F/R, respectively. The resulting PCR products were cloned into pGADT7 and pGBKT7 which were digested with *Xho*lI to create FgPEX22-like-AD and FgPEX4-BD as the prey vector and bait vector, respectively. A similar method was used to generate FgPEX4-AD and FgPEX22-like-BD. The resulting bait and prey vectors were confirmed by sequencing and co-transfected as pairs. To explore the key action regions of FgPEX4 and FgPEX22-like, truncated cDNA of *FgPEX4* and *FgPEX22-like* were amplified with their respective primers (Supplementary Table 2). These shorter PCR products were cloned into pGADT7 to construct different FgPEX22-like (a–b)-ADs as the prey vector. The same method was used to create FgPEX4 (c–d)-BDs as bait vectors. The truncated bait and prey vectors were also confirmed by sequencing and were co-transfected into yeast in various combinations (Supplementary Table 2). The interaction between pGBKT7-53 (BD-3) and pGADT7-T (AD-1) was used as a positive control.

For co-immunoprecipitation assays, the intracellular region of *FgPEX4* and *FgPEX22-like* were PCR-amplified with the primer pairs 4-GFP-F/R and 22-Flag-F/R (Supplementary Table 2), and then inserted into the vector pFL2 and pFL7 which were digested with *Xho*l I respectively. The resulting fusion constructs PEX4-GFP and *FgPEX22-like*-3 × FLAG were verified by DNA sequencing. Constructs PEX22-like-3 × FLAG and PEX22-like-3 × FLAG with PEX4-GFP were transfected into strain PH-1. For transformant selection, G418 (Geneticin) was added at a final concentration of 200 mg/mL. Total proteins were extracted and incubated with GFP beads as previously reported (Li et al., 2017). Total proteins and proteins eluted from the GFP beads (elution) (Kangti Life Technology Co., Ltd., KTSM1334) were analyzed by western blotting using monoclonal anti-FLAG antibody (Abimate medical technology (Shanghai) Co., Ltd., PA9020S) and anti-GFP antibody (Abimate medical technology (Shanghai) Co., Ltd., PA9056S), accordingly. The results were visualized using the enhanced chemiluminescent (ECL) detection system.

### Generation of *FgPEX22-Like* Gene Deletion and the Δ*PEX22-Like* Complementation Strain

The mutants were generated using the split-marker method (Wang et al., 2011). For the *FgPEX22-like* gene (FGSG_11970), the sequence was obtained from the *F. graminearum* database. The upstream (931 bp) and downstream (855 bp) regions flanking the gene were PCR-amplified using primer pairs AF/AR and BF/BR, respectively (Supplementary Table 2). The plasmid pCB1003 harbored hygromycin B resistance gene (*HPH*) and the primer pair HPH-F/HPH-R was used to amplify *HPH* (Supplementary Table 2). A fusion cassette containing the *FgPEX22-like* flanking sequences and the *HPH* gene was transfected into protoplasts of PH-1 to generate the Δ*PEX22-like* mutant (Catlett et al., 2003). Southern blot assay for *FgPEX22-like* deletion mutants was performed using the digoxigenin (DIG)-labeled probe and the High Prime DNA Labeling and Detection Starter Kit I (Roche Diagnostics, Mannheim, Germany), as instructed by the manufacturer. Hybridization was performed using a DIG-labeled specific probe (Supplementary Figure 1).

To prepare the complementation strain, a 2,674 bp fragment containing the full-length *FgPEX22-like* gene sequence and its promoter sequence was PCR-amplified using the primer pair 22CF/22CR (Supplementary Table 2) and then amplimer inserted into pYF11 using the yeast *in vivo* recombination approach (Bruno et al., 2004; Zhou et al., 2011). The recombinant plasmid pYF11-*PEX22-like* was transfected into protoplasts of theΔ*PEX22-like* mutant to produce Δ*PEX22-like* complementation (Δ*PEX22-like* -C) strains, which were identified by PCR analysis (Supplementary Figure 1).

To generate the *FgPEX22-like* and *FgPEX4* double-knockout mutants, a *FgPEX22-like* gene replacement construct was generated with the G418 amplified from pFL2 (Zhou et al., 2011) and transfected into the FgPEX4 mutant Δ*PEX4* (Table 1). Transformants resistant to both hygromycin and G418 were screened by PCR analysis (Jiang et al., 2015).

**TABLE 1:** Conidiation, conidial germination, DON production and relative expression level of *TRI* genes in PH-1, Δ*PEX22-like* and ΔΔ*PEX4/22-like* mutants.

Strain	Conidiation*	Germination (%)**	DON	Relative expression level^‡^		
	(10^6^ conidia/mL)		Production (ppm)^†^	*Tri5*	*Tri6*	*Tri10*
PH-1	21.33 ± 0.92^a^	95.07 ± 0.27^a^	314.1 ± 10.1^a^	1.00 ± 0.03^a^	1.00 ± 0.03^a^	1.00 ± 0.02^a^
Δ*PEX22-like*	16.00 ± 0.29^b^	70.20 ± 0.87^b^	29.2 ± 3.6^b^	0.51 ± 0.02^b^	0.16 ± 0.01^b^	0.52 ± 0.14^b^
ΔΔ*PEX4/22-like*	3.78 ± 0.26^c^	65.38 ± 0.47^b^	23.5 ± 2.9^b^	0.57 ± 0.03^b^	0.18 ± 0.02^b^	0.46 ± 0.04^b^

*Number of conidia in 100 mL of carboxymethylcellulose (CMC) cultures were examined after incubation for 5 days.

**Conidia were incubated in YEPD medium at 25°C for 6 h.

*^†^HPLC-MS/MS analysis of DON produced in the PH-1 and mutants.*

*^‡^Expression levels of several genes at the level of transcription. The relative expression level of GAPDH gene was used as an internal control. The gene expression in PH-1 was set to 1.0 (P < 0.05).*

*The different letter on the bars for each treatment indicates significant difference at P < 0.05 by Duncan’s multiple range test.*

### Conidiation, Germination, and Sexual Reproduction Assays

For conidiation assays, strains were inoculated into CMC medium as previously described (Hou et al., 2002). The chemical compound 4′,6-diamidino-2-phenylindole (DAPI, 10 μg/mL) and calcofluor white (CFW, 1 μg/mL) were used to stain nuclei and septa of conidia, respectively. After staining, images were captured using a fluorescence microscope (Eclipse 90i, Nikon). To determine the germination rate of conidia, freshly harvested conidia were transferred to the sterile YEPD medium (yeast extract, 10.0 g; peptone, 20.0 g; glucose, 20.0 g; and distilled water to make up the volume to 1,000 mL) for 6 h and observed under a fluorescent microscope (Eclipse 90i, Nikon). For sexual reproduction assays, 7-day-old aerial hyphae growing on specific sporulation medium were compressed in 1 mL sterile 2.5% Tween 60 solution as previously described (Bowden and Leslie, 1999; Jenczmionka et al., 2003). Perithecium formation was examined after 2–3 weeks of incubation at 25°C.

### Plant Infection and Deoxynivalenol Production Assays

Plant infections were assayed on wheat heads and corn silks. Susceptible wheat cultivar Jimai 22 was used in wheat infection assays and was sprayed with a conidial suspension (2 × 10^5^ spores/mL) collected from 5-day-old liquid CMC medium (Qin et al., 2015). Wheat heads were photographed and assayed 14 dpi. Fresh corn silks were infected with hyphal plugs, incubated at 25°C, and examined at 5 dpi (Seong et al., 2005; Gale et al., 2007). To determine DON production, we inoculated three mycelial plugs from each strain into 5 g healthy and aseptic rice grains. After incubating at 25°C, DON was extracted at 20 dpi as described previously and quantified using a liquid chromatography-mass spectrometer/mass spectrometer (HPLC–MS/MS) system (AB Sciex 5500) as previously described (Mirocha et al., 1998).

### Analysis of Fatty Acid Utilization

The carbon source utilization was evaluated using minimal medium containing various carbon sources in lieu of sucrose. The following concentrations were used: 2.5 mM myristic acid (C14), 2.5 mM palmitic acid (C16), 2.5 mM oleic acid (C18), and 2.5 mM erucic acid (C22) as previously described (Leslie and Summerell, 2007). Emulsifier NP40 was added to the minimal medium containing palmitic acid, oleic acid, and erucic acid. Colony diameters were measured after 3.5 days incubation at 25°C.

### Light Microscopy and Transmission Electron Microscopy Observations

Lipid droplets (LD) in hyphae were stained using Nile red (50 μg/mL) as reported previously (Lu et al., 2009). Hyphae grown on PDA plates at 25°C for 3 days were collected and subjected to ultrastructural analysis. The collected fungal mass was treated and examined by transmission electron microscopy (TEM) (JEM-1400 Plus, JEOL, Tokyo, Japan).

For subcellular localization of FgPEX4, peroxisome membrane protein 70 (PMP70), and Woronin body protein hex1 (HEX1), coding sequences of *FgPEX4*, *FgPMP70*, and *FgHEX1* were PCR-amplified using the primer pair 4-GFP-F/R, PMP70-GFP-F/R, and HEX1-GFP-F/R, respectively (Supplementary Table 1). The amplimers were then inserted into pYF11 using the yeast *in vivo* recombination approach (Bruno et al., 2004; Zhou et al., 2011). The recombinant plasmids were transfected into protoplasts of the WT and mutant strains. The transformants were verified by PCR analysis using the appropriate primers. Transformants were observed using a fluorescence microscope.

### ROS Detection

The ROS generated in the hyphae of PH-1 and Δ*PEX22-like* strains was assessed using NBT (nitroblue tetrazolium chloride) following the growth of the strains on CM at 25°C for 3 days. Each plate was then stained with 20 mL of 0.2% NBT solution and incubated in the dark at 28°C for 45 min. The liquid stain was drained from the plates, which were then washed with ethanol. The plates were incubated again for 45 min in the dark at 28°C prior to imaging.

### Quantitative Real-Time PCR

Total RNA was isolated from hyphae of the WT, mutant, and complementation strains using TransZol Up (TransGen Biotech, Beijing, China). The quantitative real-time PCR (qRT-PCR) experiments were performed as the manufacturer’s instructions (Vazyme Biotech Co., Jiangsu, China). The glyceraldehyde 3-phosphate dehydrogenase (GAPDH) gene of *F. graminearum* was used as the internal control. Relative expression levels for each gene were calculated using the 2^–ΔΔ*CT*^ method (Livak and Schmittgen, 2001).

### Statistical Analysis

Each experiment was performed in three individual replicates. Data are presented as mean ± standard error values and the differences among variables were analyzed using Duncan’s multiple range test. Results with *p* < 0.05 were considered statistically significant.

## Results

### Identification of *FgPEX22-**Like*** in *Fusarium graminearum*

The homologs of PEX22 (FGSG_11970) were identified using the Fusarium genome database^1^. The predicted gene was 1,631 bp, encoding 363 amino acids. FgPEX22-like shared only 14.34% identity with *C. orbiculare* and 17.93% identity with *S. cerevisiae* PEX22. Therefore, we named the gene *FgPEX22-like*. SMART-PFAM analysis revealed the protein encoded by *PEX22-like* contained a transmembrane domain and a possible PEX4 interacting region similar to that in *C. orbiculare* and *S. cerevisiae* (Supplementary Figure 1A). The deduced FgPEX22-like protein was distributed in one branch in the phylogenetic tree along with other fungal PEX22 proteins (Supplementary Figure 1B).

### *FgPEX22-Like* Interacted With FgPEX4 in Yeast Two-Hybrid and Co-immunoprecipitation Assays

**FIGURE 1:**
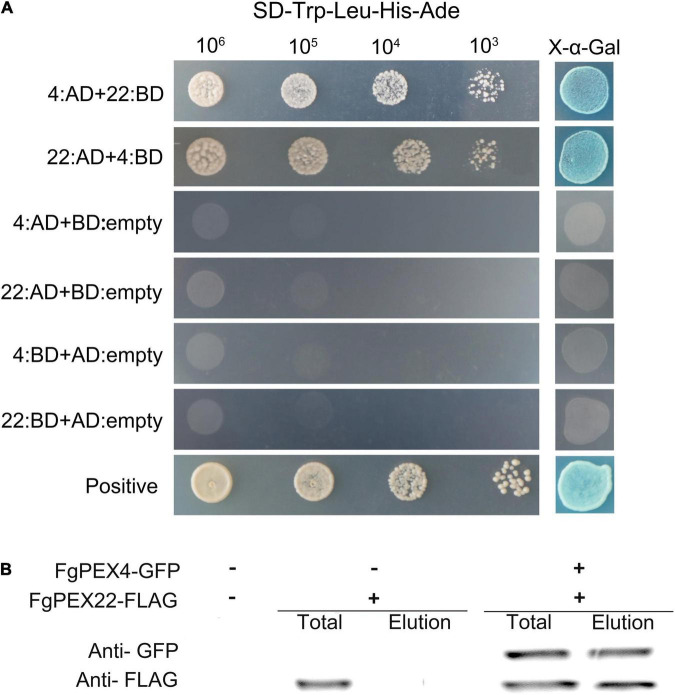
Interaction of FgPEX4 and FgPEX22-like. **(A)** Y2H assays were performed to detect interactions between FgPEX4 and FgPEX22-like. All constructed yeast transformants were tested for growth on SD-Leu-Trp-His-Ade and evaluated for β-galactosidase (LacZ) activity. The interaction of AD-1 and BD-3 was used as the positive control. All transformants were diluted to different concentrations (cells/ml) and plated onto SD-Leu-Trp-His-Ade media. 22 and 4 referring to FgPEX22-like and FgPEX4 respectively. **(B)** Co-immunoprecipitation assays. Total proteins were extracted from transformants co-expressing PEX4–GFP and PEX22–3 × FLAG constructs. The proteins were then eluted from the anti-GFP M2 beads (elution). The immunoblots were incubated with monoclonal anti-FLAG or anti-GFP antibody, as indicated.

To investigate whether FgPEX22-like interacted with FgPEX4 in *F. graminearum*, a yeast two-hybrid assay was used. Yeast transformants expressing binding domain (BD)-FgPEX4 as the bait and the activation domain (AD) pGADT7 alone as the prey, could not grow on the Sabouraud dextrose (SD)-Leu-Trp-His-Ade plates, excluding the possibility of self-activation. This was also true for the reverse situation. The sets of interacting pairs, BD-FgPEX4 and AD-FgPEX22-like or AD-FgPEX4 and BD-FgPEX22-like, were found to bind with each other (Figure 1A).

To further verify these results, the intracellular region of FgPEX4 was fused with pFL2 [PEX4-green fluorescent protein (GFP)] and co-transfected into *F. graminearum* strain PH-1 with the FgPEX22-like-3 × FLAG fusion construct. In western blot analysis of total proteins from the strain containing PEX22-3 × FLAG, a 40-kDa band was detected using the anti-FLAG antibody, while the elution proteins were not detected. This showed the beads had been washed prior to protein elution and that there were no other contaminants, confirming the subsequent results were reliable. In the transformants co-expressing FgPEX22-like-3 × FLAG and FgPEX4-GFP, 19-kDa and 40-kDa bands were detected using the anti-FLAG and anti-GFP antibodies, respectively (Figure 1B). Therefore, FgPEX22-like interacted with FgPEX4 in *F. graminearum*. Taken together, these results demonstrated the two proteins could interact directly with each other.

Based on the predicted secondary structure and domain structure, we explored the key functional regions of FgPEX4 and FgPEX22-like. FgPEX22-like contained an N-terminal transmembrane (TM) fragment and a large region spanning an unknown fold that was exposed in cytosol. Several truncated variants of FgPEX22-like were established and their interactions with FgPEX22-like and FgPEX4 were evaluated using the yeast two-hybrid system. Our results showed that the region spanning residues 102–282 was essential for FgPEX4 binding; moreover, residues were part of the soluble region of FgPEX22-like. Meanwhile, we also discovered that the first 11 amino acids of FgPEX4 were not necessary for FgPEX22-like binding (Supplementary Figure 2).

### *FgPEX22-Like* Acted as a Rivet Protein of FgPEX4

**FIGURE 2:**
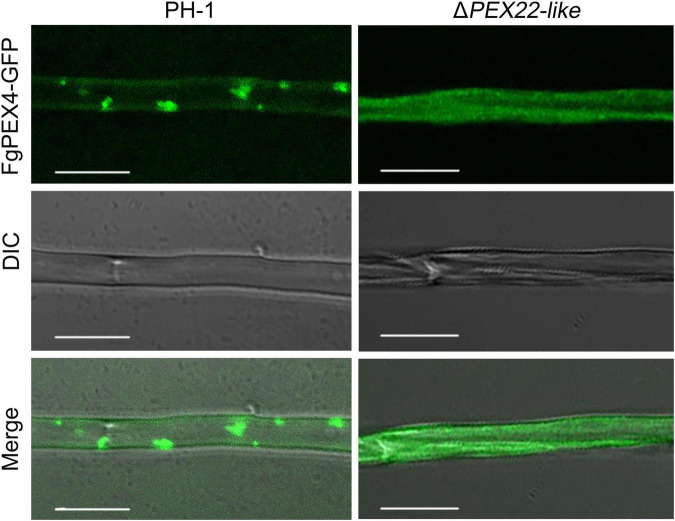
Subcellular localization of FgPEX4 in *F. graminearum*. Mycelium of all the strains were collected from 5-day-old complete medium (CM) plates and observed using confocal fluorescence microscopy. observe, but FgPEX4-GFP in Δ*PEX22-like* exhibited a completely cytoplasmic distribution. Bar = 5 mm.

To further understand the relationship between the two proteins, we compared the subcellular localization of wild-type (WT) FgPEX4 to that of mutant strains. Interestingly, the results showed that a deficiency of *FgPEX22-like* could result in abnormal subcellular localization of FgPEX4. FgPEX4-GFP fusions was distributed in punctate patterns in transformed PH-1, but were dispersed in the cytoplasm of the *PEX22-like* deletion mutant Δ*PEX22-like* (Figure 2). These results indicated FgPEX22-like was necessary for the location of FgPEX4 and that it functioned as a rivet of FgPEX4.

### *FgPEX22-Like* Was Involved in Sexual and Asexual Reproduction

To elucidate the biological functions of FgPEX22-like in *F. graminearum*, we generated the single-gene knockout mutant Δ*PEX22-like* and the double-gene knockout mutant ΔΔ*PEX4/22-like* by split-marker polymerase chain reaction (PCR) and protoplast transformation. A schematic diagram depicting the strategy used to create the *FgPEX22-like* gene deletion mutant and molecular analysis of Δ*PEX22-like* is shown in Supplementary Figure 3. The pYF11-PEX22-like complementation construct was generated by PCR amplification using the primer pair 22hfF/22hfR (Supplementary Table 1), followed by transformation of Δ*PEX22-like* mutant protoplasts to produce the Δ*PEX22-like* complementation strain Δ*PEX22-like*-C.

**FIGURE 3:**
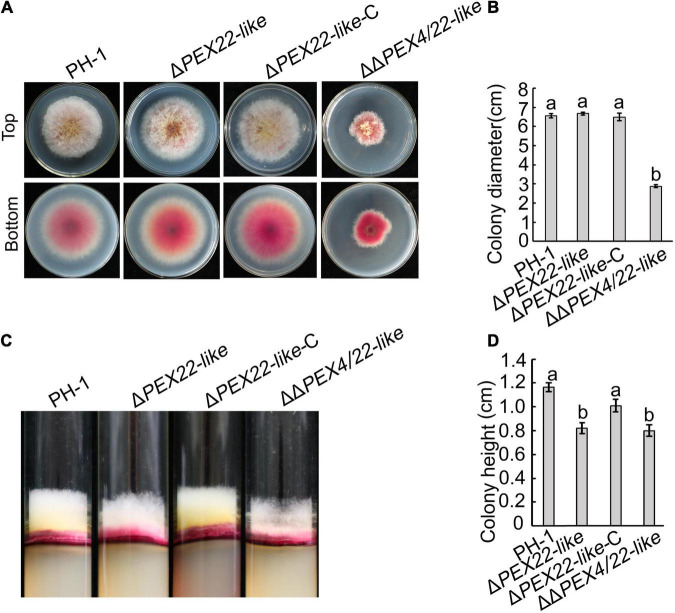
Effects of *FgPEX22-like* on hyphal growth in *F. graminearum*. **(A)** Growth of *F. graminearum* strain PH-1, Δ*PEX22-like*, Δ*PEX22*-*like*-C, and ΔΔ*PEX4/22-like* strains on potato dextrose agar (PDA) plates. The four strains were imaged after 3 days. **(B)** Colony growth by PH-1, Δ*PEX22-like*, Δ*PEX22-like* -C, and ΔΔ*PEX4/22-like* strains cultured on PDA plates. **(C)** Height of aerial mycelium produced by PH-1, Δ*PEX22-like*, Δ*PEX22-like*-C, and ΔΔ*PEX4/22-like* strains. The four strains were culture in transparent test tubes containing PDA medium and imaged after 5 days. **(D)** Colony height of PH-1, Δ*PEX22-like*, Δ*PEX22-like* -C, and ΔΔ*PEX4/22-like* strains cultured in tubes.

No obvious vegetative growth or colony morphology defects were observed for Δ*PEX22-like* compared to that of the WT strain. The *FgPEX4* and *FgPEX22-like* double mutants exhibited a significant reduction in growth rate compared to that of the *FgPEX22-like* single mutant and produced compact colonies with limited aerial hyphae (Figures 3A–D). These results suggested FgPEX22-like was not involved alone in the regulation of hyphal growth of *F. graminearum*.

**FIGURE 4:**
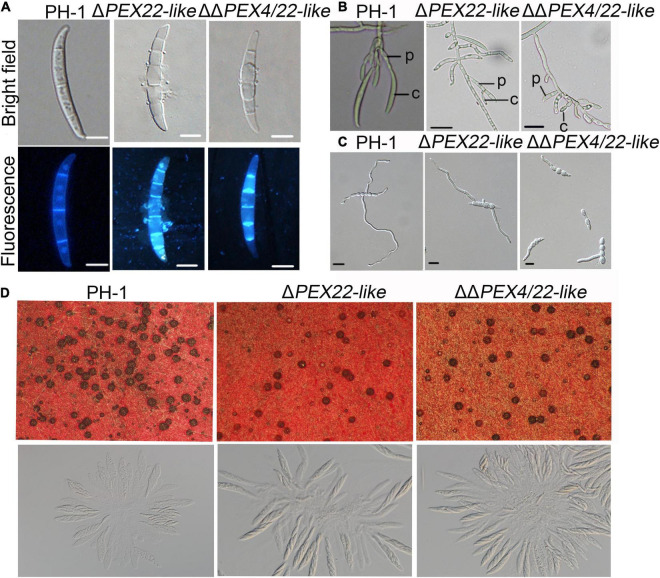
The Δ*PEX22-like* and ΔΔ*PEX4/22-like* mutants exhibit defects in conidial morphology, conidiogenesis, germination, and sexual reproduction. **(A)** Conidia of *F. graminearum* strain PH-1, Δ*PEX22-like*, and ΔΔ*PEX4/22-like* were incubated in CMC medium without any agar for 3 days and observed using differential interference contrast (DIC) and fluorescence microscopy. Conidia were stained with 1 μg/mL calcofluor white (CFW) and 10 μg/mL of DAPI. Bar = 10 μm. **(B)** Conidiogenesis of PH-1, Δ*PEX22-like*, and ΔΔ*PEX4/22-like.* C, conidium; P, phialide. Bar = 20 μm. **(C)** Conidial germination of PH-1, Δ*PEX22-like*, and ΔΔ*PEX4/22-like*. Conidia were incubated in YEPD without agar and germination was imaged 6 h post incubation. Bar = 10 μm. **(D)** Defect of Δ*PEX22-like* and ΔΔ*PEX4/22-like* in sexual reproduction.

When assayed for conidiation in carboxymethyl cellulose (CMC) medium cultures, Δ*PEX22-like* and ΔΔ*PEX4/22-like* were reduced by 25.0 and 82.3%, respectively, compared with that of the WT strain (Table 1). In addition, conidia of the mutants exhibited normal conidium morphology, displaying conidia that lacked intracellular content and devoid of nuclei (Figure 4A). Phialides of the Δ*PEX22-like* and ΔΔ*PEX4/22-like* mutants were rarely clustered together, unlike those of PH-1 (Figure 4B), which may have been directly responsible for reduced conidiation in the Δ*PEX22-like* mutant. When incubated in yeast extract peptone dextrose (YEPD) medium, the conidium germination of Δ*PEX22-like* and ΔΔ*PEX4/22-like* at 6-h post-incubation was reduced by 26.2 and 31.2%, respectively, compared with that of PH-1 and the germ tubes were shorter than those of PH-1 (Figure 4C and Table 1). The ability of the *FgPEX22-like* mutants to undergo sexual reproduction was also investigated. We found the number of perithecia for Δ*PEX22-like* and ΔΔ*PEX4/22-like* were reduced by 65.0 and 56.6%, respectively, compared with that of the WT strain at 2-wk post-fertilization, and the ascocarp is no difference between them (Figure 4D). Together, these results demonstrated that *FgPEX22-like* played vital roles in conidiation, conidial germination, and sexual reproduction.

### *FgPEX22-Like* Was Important for Virulence and Deoxynivalenol Production

**FIGURE 5:**
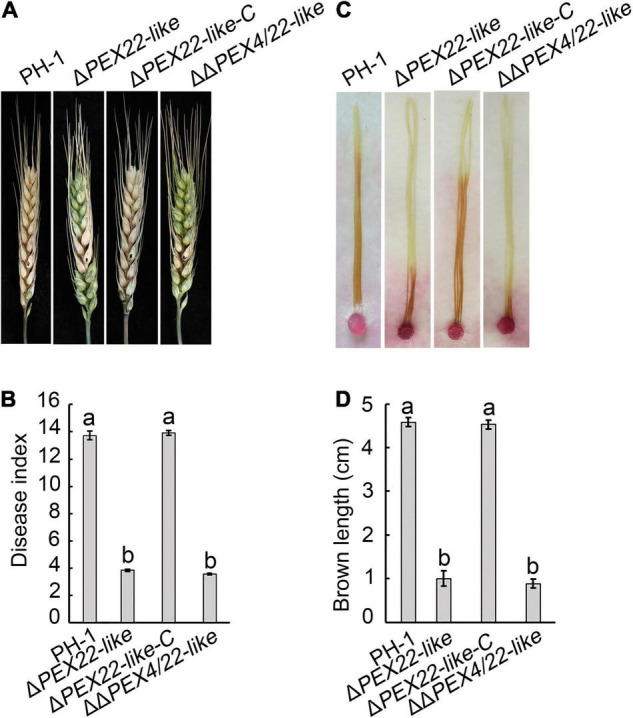
Pathogenicity defects in the *FgPEX22-like* deletion mutant. **(A)** Typical symptoms caused by *F. graminearum* strain PH-1, Δ*PEX22-like*, Δ*PEX22-like*-C, and ΔΔ*PEX4/22-like* on wheat heads. Each inflorescence of wheat cultivar Jimai 22 was inoculated with 10 μL conidial suspension (2 × 10^5^ conidia/mL) and pathogenesis was recorded at 14 days post inoculation (dpi). **(B)** Disease indices of three strains determined at 14 dpi. More than 30 wheat heads were examined in each replicate. The error bars represent the standard errors of the means. **(C)** Brown necrosis caused by Δ*PEX22-like*, Δ*PEX22-like -*C, and ΔΔ*PEX4/22-like* on corn silks. Mycelial plugs were placed on one side of the corn silks, which were arrayed on wet filter paper to maintain high humidity. Photographs were taken 5 dpi at 25°C. **(D)** Length of brown necrotic tissue infected by three strains were determined 5 dpi. More than 30 corn silks were examined in each replicate.

The pathogenicity assays were performed to ascertain the effect of Δ*PEX22-like* on flowering wheat heads and corn silks. Wheat heads inoculated with either PH-1 or the Δ*PEX22-like* -C strain presented typical scabs, spreading from the inoculated spikelets to almost the entire head by 14 days post inoculation (dpi). In contrast, wheat heads inoculated with the Δ*PEX22-like* mutant presented scabs mainly on or near the inoculated spikelets (Figure 5A). The disease index of Δ*PEX22-like* and ΔΔ*PEX4/22-like* exhibited approximately a 71.9 and 74.0% reduction, respectively, compared with that of PH-1 (Figure 5B). In corn silk infection, the extended length of brown lesions caused by PH-1 and the Δ*PEX22-like*-C strain were longer than those of Δ*PEX22-like* and ΔΔ*PEX4/22-like*. The ability of Δ*PEX22-like* or ΔΔ*PEX4/22-like* to infect corn silk was only 1/5 that of the WT strain at 5 dpi (Figures 5C,D).

We also assayed Δ*PEX4* and ΔΔ*PEX4/22-like* for DON production. We found that infected rice seeds inoculated with the Δ*PEX22-like* mutant produced significantly less DON than that of PH-1 or the Δ*PEX22-like-*C strain (Table 1). These results indicated *FgPEX22-like* had an important role in pathogenicity and DON production.

### *FgPEX22-Like* Deficiency Caused Abnormal Organelle Development

**FIGURE 6:**
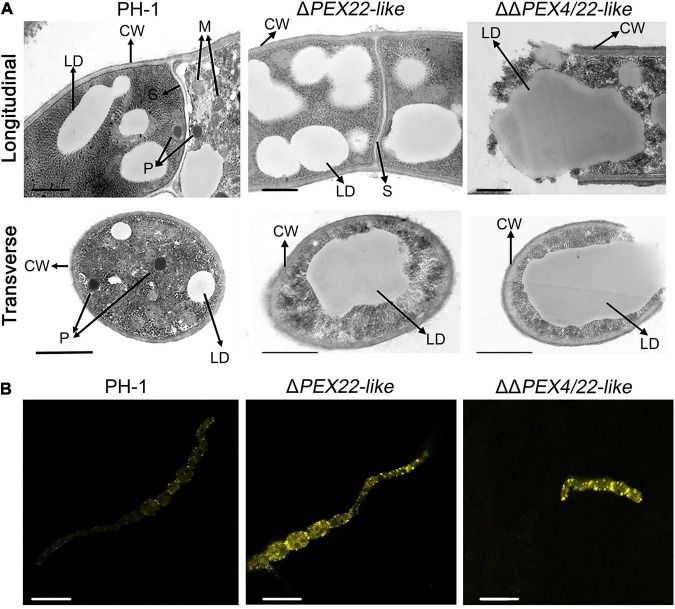
Ultrastructure and lipid droplets in *F. graminearum* and the Δ*PEX22-like* deletion mutant. **(A)** Ultrastructure of *F. graminearum* strain PH-1 and mutants Δ*PEX22-like* and ΔΔ*PEX4/22-like*. CW, cell wall; M, mitochondria; S, septum; P, peroxisome; W, Woronin body; LD, lipid droplet. Hyphae grown on PDA plates for 3 days were analyzed by TEM. Bar = 1 μm. **(B)** Accumulated LDs in hypha and conidium of the Δ*PEX22-like* mutant were stained using Nile red. Images were acquired using a laser scanning confocal microscope. Bar = 10 μm.

To determine the effect of FgPEX22-like on *F. graminearum* organelles, the ultrastructure of PH-1 and Δ*PEX22-like* was evaluated using TEM. Spherical peroxisomes were observed in the periphery of mycelial cells of PH-1, but not Δ*PEX22-like*. In addition, Woronin bodies, which were present around the mycelial septum in the WT strain, were absent in the Δ*PEX22-like* mutant (Figure 6A). Besides, Compared with WT strain, Δ*PEX22-like* accumulated more lipid droplets. And Nile Red staining revealed that the WT strain degraded most of the lipid droplets, while mutant strains still contained numerous bright lipid droplets (Figure 6B).

**FIGURE 7:**
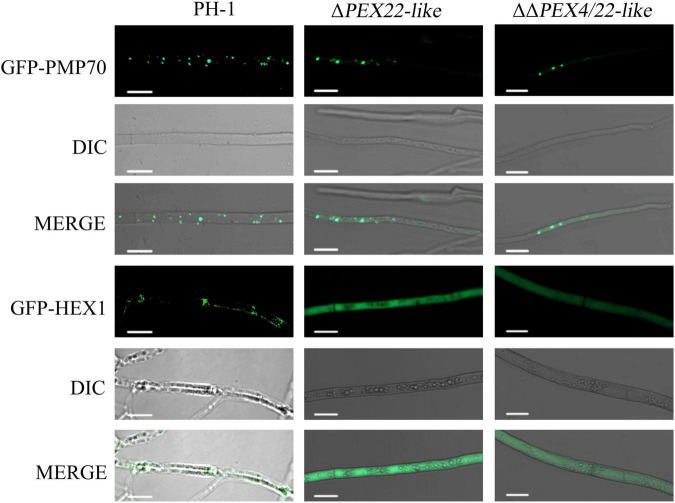
Subcellular localization of FgPMP70 and FgHEX1 in *F. graminearum*. Subcellular localization of FgPMP70 and HEX1. Mycelium of all the strains were harvested from 5-day-old complete medium (CM) plates and detected using confocal fluorescence microscopy. GFP-PMP70 was predominantly present in a punctate pattern (peroxisomal distribution) in the wild-type and Δ*PEX22-like*. Bar = 5 mm. GFP-HEX1 was bimodally distributed in a punctate pattern (peroxisomal distribution) in the wild-type, but GFP-HEX1 in Δ*PEX22-like* was completely cytoplasmically distributed. Bar = 10 mm.

To confirm the existence of peroxisome and Woronin body, GFP fusion constructs with the Woronin body protein HEX1 or PMP70 were transfected into the WT and mutant strains and evaluated using laser-scanning confocal microscopy. For PMP70-GFP, Δ*PEX22-like* displayed a green punctate distribution in hyphal cells similar to that in the WT strain (Figure 7). However, the fusion of GFP with the Woronin body protein HEX1 resulted in a punctate distribution in the WT strain while being dispersed in the cytoplasm of Δ*PEX22-like* (Figure 7). These results indicated FgPEX22-like was indispensable for maintaining Woronin bodies, but not peroxisomes.

In addition, based on the TEM observations, the mutant accumulated more lipid drops, indicating the utilization rate of cellular lipid drops in Δ*PEX22-like* was slower than that in PH-1. Further verification was carried out by performing Nile red staining. Brighter and larger lipid droplets were observed in Δ*PEX22-like* compared to that of the WT strain. This was consistent with the TEM findings.

### Deletion of *FgPEX22-Like* Altered the Cell Wall Integrity of *Fusarium graminearum*

To investigate whether FgPEX22-like participated in environmental stress responses, we examined the sensitivity of mutants to the cell wall-damaging agent Congo red and the cell membrane-damaging agent sodium dodecyl sulfate (SDS). We found that Δ*PEX22-like* exhibited increased sensitivity to both SDS and Congo red compared to that of PH-1 (Supplementary Figures 4A,B). To verify these results, hyphae were treated with lysozyme and driselase. After incubation at 30°C for 30 min, hyphae of the Δ*PEX22-like* mutant were almost completely digested and had released abundant numbers of protoplasts, whereas few protoplasts were observed among hyphae of the PH-1 and Δ*PEX22-like -*C strains (Supplementary Figure 4C). Together, these results illustrated that deletion of *FgPEX22-like* resulted in reduced cell wall integrity in *F. graminearum.*

### *FgPEX22-Like* Mutant Was More Sensitive to ROS and Involved in Lipid Metabolism

To investigate whether *FgPEX22-like* was involved in the response to oxidative stress, the tolerance of Δ*PEX22-like* to ROS was measured. The radial growth of Δ*PEX22-like* and ΔΔ*PEX4/22-like* were inhibited by 60.0 and 56.8%, respectively, when exposed to 20 mM H_2_O_2_, which was greater than that of PH-1 at 42.7% (Supplementary Figures 5A,B). Cellular ROS production in the hyphae was qualitatively analyzed by staining with nitroblue tetrazolium (NBT). The assay showed that the staining of Δ*PEX22-like* and ΔΔ*PEX4/22-like* hyphae were darker than those of PH-1 (Supplementary Figure 5C). Taken together, these results demonstrated the capacity of Δ*PEX22-like* and ΔΔ*PEX4/22-like* to eliminate ROS was decreased.

**FIGURE 8:**
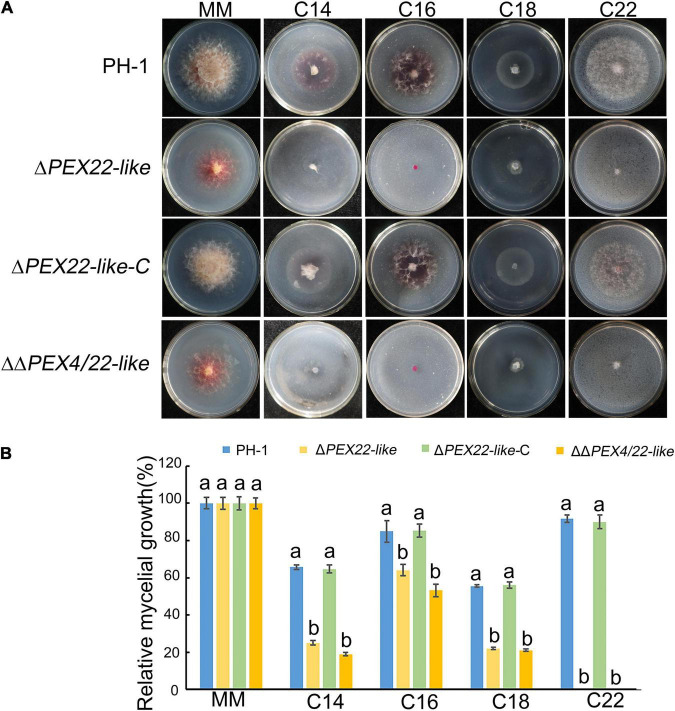
Relative mycelial growth of strains on different carbon sources. **(A)** *F. graminearum* strain PH-1, Δ*PEX22-like*, Δ*PEX22-like-*C, and ΔΔ*PEX4/22-like* were cultured for 3.5 days on minimal medium (MM) containing 2.5 mM myristic acid (C14), 2.5 mM palmitic acid (C16), 2.5 mM oleic acid (C18), or 2.5 mM erucic acid (C22) as the sole carbon source. **(B)** Relative mycelial growth of PH-1, Δ*PEX22-like*, Δ*PEX22-like-*C, and ΔΔ*PEX4/22-like* on MM with different carbon sources.

To determine the effect of deleting *FgPEX4* on fatty acid utilization, vegetative growth was assessed using minimal medium with different fatty acids as sole carbon sources. The fatty acids tested included long-chain fatty acids myristic acid (C14), palmitic acid (C16), and oleic acid (C18), and very long-chain fatty acid erucic acid (C22). After incubation with the particular fatty acids for 3 days, the radial growth of the mutants was significantly reduced on the media containing the long-chain fatty acids and very long-chain fatty acids as the sole carbon sources (Figures 8A,B). These results indicated that deletion of *FgPEX22-like* in *F. graminearum* resulted in a defect in fatty acid metabolism.

## Discussion

Previous studies have analyzed the whole-genome sequences of 17 fungal species and found that PEX22 is less conserved than that of most peroxins (Kiel et al., 2006). In the current study, we identified a new peroxin in *F. graminearum*, FgPEX22-like, which has a PEX4-binding site and can directly interact with FgPEX4. Deletion of *FgPEX*2*2-like* resulted in mislocation of FgPEX4. In addition, we found that FgPEX22-like was involved in the regulation of development, carbon source utilization, cell wall integrity, and pathogenicity of *F. graminearum.* To our knowledge, this is the first report of a PEX22 homolog playing an important role in *F. graminearum.*

A previous study found that yeast pex22 mutant can be fully complemented by FAM1, which is a functional ortholog of PEX22 in *C. orbiculare* (Kubo et al., 2015). The peroxin described in this study shared 14.34% identity with *C. orbiculare* and named FgPEX22-like. Although there is low sequence similarity between FgPEX22-like, FAM1 and ScPEX22, FgPEX22-like is structurally similar to other PEX22 proteins with a single predicted TM domain near the N terminus and a possible PEX4 binding interface near the C terminus. In *S. cerevisiae* and *Arabidopsis*, PEX4 and PEX22 can interact with each other directly (Zolman et al., 2005; Williams et al., 2012). To determine whether FgPEX22-like functioned similar to that of PEX22 in *S. cerevisiae* and *Arabidopsis*, a yeast two-hybrid system and immunoprecipitation were employed. The results showed that FgPEX22-like could interact directly with FgPEX4 in *F. graminearum*.

To explore the relationship between FgPEX22-like and FgPEX4, the subcellular localization of FgPEX4 in the WT and *FgPEX22-like* gene deletion strain was evaluated. We found FgPEX4 in the *FgPEX22-like* deletion strain mislocated, which suggested that FgPEX22-like anchored FgPEX4 on the peroxisomal membrane and acted as a rivet protein for PEX4. These results were consistent with PEX22 in *Pichia pastoris* in which PEX4 is unstable in a Δ*PEX22* strain (Koller et al., 1999). The results illustrated that FgPEX22-like is the homologous protein of PEX22 in *F. graminearum* and is an important peroxin at the peroxisomal membrane that recruits and holds PEX4 at this location.

In *S. cerevisiae*, PEX22 plays an important role in many developmental processes (Negoro et al., 2018). In this study, we evaluated FgP*EX22-like*, the *FgPEX22-like* mutant Δ*PEX22-like*, and the *FgPEX4* double-knockout mutant ΔΔ*PEX4/22-like*. The results showed that *FgPEX22-like* on its own lacked any notable phenotype regarding hyphal growth, but it enhanced *PEX4* mutant defects. This suggested that FgPEX4 and FgPEX22-like interact with each other at the molecular level and FgPEX22-like contributes to peroxisome function.

Herein, the Δ*PEX22-like* mutant exhibited defects in sexual and asexual reproduction, producing less perithecium and conidiation formation and abnormal conidiation morphologies. Similar results show that mutants with deletion of any of seven other *FgPEX* genes, *FgPEX1*, *FgPEX2*, *FgPEX4*, *FgPEX5*, *FgPEX6*, *FgPEX7*, and *FgPEX12*, form normal perithecia with mature ascospores; however, the production of perithecia in these mutants is reduced compared to that in the WT strain (Min et al., 2012; Zhang et al., 2019a; Wang et al., 2020). In contrast, in *Podospora anserine*, mutants with *PEX1*, *PEX4*, *PEX6*, *PEX8*, *PEX22*, or *PEX26* deleted are sterile (Suasteolmos et al., 2018). Taken together, we can conclude that FgPEX22-like contributes to the development of *F. graminearum*.

Peroxisome functions are important factors in plant infections. In *C. orbiculare*, FAM1, which encodes a woronin-body-associated PEX22, plays important roles in appressorium development and pathogenicity (Kubo et al., 2015). In this study, the Δ*PEX22-like* mutant showed a significant reduction in disease severity. In addition, similar to FgPEX1, FgPEX2, FgPEX3, FgPEX4, FgPEX10, FgPEX12, FgPEX13, FgPEX14, and FgPEX33, we found that toxin production, which is known to be essential for the virulence of *F. graminearum* (Desjardins et al., 1993; Chen et al., 2018; Kong et al., 2019; Zhang et al., 2019a,b; Wang et al., 2020), was obviously reduced in the *FgPEX22* deletion strain. Similarly, most peroxins in *M. oryzae*, except for PEX5, have critical roles in virulence (Wang et al., 2007, 2019; Goh et al., 2011; Chen et al., 2016; Li et al., 2017). In *Alternaria alternata* and *Colletotrichum gloeosporioides*, peroxisomes are also important for the biosynthesis of AK-toxin and plant infection (Imazaki et al., 2010; Zhao et al., 2020). These results indicate that different peroxins have different regulatory mechanisms in regulating pathogenicity, and FgPEX22-like is an important pathogenic factor of *F. graminearum*.

Interestingly, the results of electron microscopy showed the microstructure of Δ*PEX22-like* displayed an abnormal morphology in the following four aspects. First, the peroxisome structures were absent in the *FgPEX22-like* deletion mutant. In humans, mutations in the AAA-complex *PEX26*, *PEX1*, and *PEX6* result in a decrease in peroxisome number and function (Law et al., 2017). To verify the presence or absence of peroxisome structures in the *FgPEX22-like* deletion mutant, we determined the localization in the mycelia of a known peroxisomal membrane protein, PMP70. The results showed that PMP70-GFP in the Δ*PEX22-like* mutant displayed a punctate distribution in hyphal cells, similar to that in PH-1, indicating that the deletion of *FgPEX22-like* had no effect on the existence of peroxisomes.

Second, Woronin bodies were also absent in the *FgPEX22-like* deletion mutant. To verify this result, we determined the localization of the Woronin body protein HEX1 in the mycelia. The results showed that HEX1-GFP was dispersed uniformly in the cytoplasm of Δ*PEX22-like*. This phenotype was in agreement with the functions of FgPEX1, FgPEX2, and FgPEX10 in the biogenesis of Woronin bodies. In addition, MoPEX11, MoPEX19, and MoPEX14/17 are also essential in *M. oryz*ae for the biogenesis of Woronin bodies (Li et al., 2014, 2017). Woronin body, which is derived from peroxisomes, is a characteristic organelle specifically present in filamentous ascomycetes. Woronin bodies can plug septa into intact growing hyphae to maintain hyphal heterogeneity in a fungal mycelium by impeding cytoplasmic continuity (Liu et al., 2008).

Third, deletion of *FgPEX22-like* resulted in reduced cell wall integrity, displaying considerable leakage of hyphae and conidia. This result showed that the absence of *FgPEX22-like* influenced *F. graminearum* cell wall integrity. This finding was further confirmed by the sensitivity measurement of PH-1 and Δ*PEX22* to cell wall-damaging agents and degrading enzymes. Similar results were obtained for FgPEX2, FgPEX4, MoEX5, MoPEX6, MoPEX14, MoPEX17, and MoEX19, indicating they too are essential for cell wall integrity in *F. graminearum* and *M. oryzae*, accordingly (Li et al., 2014, 2017; Zhang et al., 2019b; Wang et al., 2020). Studies have shown that the fungal cell wall glucan and chitin are derived from acetyl-CoA, which is a product of β-oxidation in peroxisomes (Ramospamplona and Naqvi, 2006). Thus, we conjectured that the damaged cell wall was related to decreased acetyl-CoA. In addition, damaged cell walls of spores and mycelia may have confirmed the functional disorders in Woronin bodies, which serve as a plug to impede cytoplasmic continuity when the mycelium is damaged to maintain hyphal heterogeneity (Tenney et al., 2000). Taken together, FgPEX22-like plays a crucial role in regulating *F. graminearum* cell wall integrity.

Finally, based on TEM observations, lipid droplets accumulated and increased in both quantity and size in the Δ*PEX22-like* mutant. Nile red staining further verified this result. Previous studies have emphasized that peroxisomes maintain a close association with lipid bodies, which constitute the intracellular storage sites of triacylglycerol and cholesterol ester (Binns et al., 2006; Farese and Walther, 2009; Beller et al., 2010). In *M. oryzae*, *F. graminearum*, and *Aspergillus flavus*, the translocation and degradation of lipid droplets are also damaged by the deletion of *MoPEX1*, *FgPEX1*, *FgPEX2*, and *AflPex5* (Deng et al., 2016; Zhang et al., 2018, 2019a; Wang et al., 2020). The deletion of *PEX6* in *Colletotrichum lagenarium* abrogates its ability to use long-chain fatty acids (Matsuzono et al., 1999). When cultured on medium using long-chain fatty acids or very long-chain fatty acids as sole carbon sources, the Δ*PEX22-like* mutant exhibited slower growth rates, indicating FgPEX22-like was involved in utilizing long-chain and very long-chain fatty acids. Taken together, these results indicate that deletion of *FgPEX22-like* results in a deficiency in the utilization of lipids and long-chain fatty acids. Combining these results, we suggest that *FgPEX22-like* plays essential roles in maintaining normal organelle development in *F. graminearum*, which probably accounts for the loss of pathogenicity in the *FgPEX22-like* mutant.

Herein, we show that FgPEX22-like encodes a peroxin protein in *F. graminearum* that interacts directly with FgPEX4 and acts as a rivet protein of FgPEX4, and the loss of FgPEX22-like leads to the abnormal subcellular localization of FgPEX4 protein. Moreover, FgPEX22-like is involved in the regulation of asexual and sexual reproduction, pathogenicity, cell wall integrity, oxidative stress, and organelle integrity. Our study has established, for the first time, the comprehensive biological functions of a homologous protein of PEX22 in *F. graminearum*.

## Data Availability Statement

The data that supports the findings of this study are available in the Supplementary Material of this article.

## Author Contributions

JY: writing—review and editing. LZ and CL: investigation and writing—original draft preparation. MW: data curation. YT: software. YL: resources. All authors contributed to manuscript revision, read, and approved the submitted version.

## Conflict of Interest

The authors declare that the research was conducted in the absence of any commercial or financial relationships that could be construed as a potential conflict of interest.

## Publisher’s Note

All claims expressed in this article are solely those of the authors and do not necessarily represent those of their affiliated organizations, or those of the publisher, the editors and the reviewers. Any product that may be evaluated in this article, or claim that may be made by its manufacturer, is not guaranteed or endorsed by the publisher.

## Footnotes

1
http://www.broadinstitute.org/annotation/genome/fusariumgroup/MultiHme.Html

## References

[B1] Albertini M. Rehling P. Erdmann R. Girzalsky W. Kiel J. A. Veenhuis M. (1997). Pex14p, a peroxisomal membrane protein binding both receptors of the two PTS-dependent import pathways. *Cell* 4 83–92. 10.1016/s0092-8674(00)80185-3 9094717

[B2] Beller M. Thiel K. Thul P. J. Jäckle H. (2010). Lipid droplets: a dynamic organelle moves into focus. *FEBS Lett.* 584 2176–2182. 10.1016/j.febslet.2010.03.022 20303960

[B3] Binns D. Januszewski T. Chen Y. Hill J. Markin V. S. Zhao Y. (2006). An intimate collaboration between peroxisomes and lipid bodies. *J. Cell Biol.* 173 719–731. 10.1083/jcb.200511125 16735577 PMC2063889

[B4] Bowden R. L. Leslie J. F. (1999). Sexual recombination in *Gibberella zeae*. *Phytopathology* 89 182–188. 10.1094/phyto.1999.89.2.182 18944794

[B5] Bruno K. S. Tenjo F. Li L. Hamer J. E. Xu J. R. (2004). Cellular localization and role of kinase activity of *PMK1* in *Magnaporthe grisea*. *Eukaryot Cell* 3 1525–1532. 10.1128/EC.3.6.1525-1532.2004 15590826 PMC539019

[B6] Catlett N. L. Lee B. N. Yoder O. C. Turgeon B. G. (2003). Split-marker recombination for efficient targeted deletion of fungal genes. *Fungal Genet. Newsl.* 50 9–11. 10.4148/1941-4765.1150

[B7] Chayakulkeeree M. Sorrell T. C. Siafakas A. R. Wilson C. F. Pantarat N. Gerik K. J. (2008). Role and mechanism of phosphatidylinositol-specific phospholipase C in survival and virulence of *Cryptococcus neoformans*. *Mol. Microbiol.* 69 809–826. 10.1111/j.1365-2958.2008.06310.x 18532984

[B8] Chen X. L. Wang Z. Liu C. (2016). Roles of peroxisomes in the rice blast fungus. *Biomed. Res. Int.* 2016:9343417. 10.1155/2016/9343417 PMC5004026 27610388

[B9] Chen Y. Zheng S. Y. Ju Z. Z. Zhang C. Q. Tang G. F. Wang J. (2018). Contribution of peroxisomal docking machinery to mycotoxin biosynthesis, pathogenicity and pexophagy in the plant pathogenic fungus *Fusarium graminearum*. *Environ. Microbiol.* 20 3224–3245. 10.1111/1462-2920.14291 29901274

[B10] Collins C. S. Kalish J. E. Morrell J. C. McCaffery J. M. Gould S. J. (2000). The peroxisome biogenesis factors pex4p, pex22p, pex1p, and pex6p act in the terminal steps of peroxisomal matrix protein import. *Mol. Cell. Biol.* 20 7516–7526. 10.1128/MCB.20.20.7516-7526.2000 11003648 PMC86304

[B11] Dean R. Jan A. L. Kan V. Pretorius Z. A. Hammond K. E. Pietro A. D. (2012). The Top 10 fungal pathogens in molecular plant pathology. *Mol. Plant Pathol.* 13 804–804. 10.1111/j.1364-3703.2012.00822.x PMC6638784 22471698

[B12] Deng S. Z. Gu Z. K. Yang N. Y. Li L. Yue X. F. Que Y. W. (2016). Identification and characterization of the peroxin 1 gene *MoPEX1* required for infection-related morphogenesis and pathogenicity in *Magnaporthe oryzae*. *Sci. Rep.* 6:36292. 10.1038/srep36292 27824105 PMC5099783

[B13] Deng Y. Qu Z. Naqvi N. I. (2013). The role of snx41-based pexophagy in *Magnaporthe* development. *PLoS One* 8:e79128. 10.1371/journal.pone.0079128 24302988 PMC3841179

[B14] Desjardins A. E. Hohn T. M. Mccormick S. P. (1993). Trichothecene biosynthesis in *Fusarium* species: chemistry, genetics, and significance. *Microbiol. Rev.* 57 595–604. 10.1128/mr.57.3.595-604.1993 8246841 PMC372927

[B15] Distel B. Erdmann R. Gould A. J. Blobel G. Crane D. I. Cregg J. M. (1996). A unified nomenclature for peroxisome biogenesis factors. *J. Cell Biol.* 135 1–3. 10.1083/jcb.135.1.1 8858157 PMC2121017

[B16] Farese R. V. Walther T. C. (2009). Lipid droplets finally get a little R-E-S-P-E-C-T. *Cell* 139 855–860. 10.1016/j.cell.2009.11.005 19945371 PMC3097139

[B17] Faust P. L. Banka D. Siriratsivawong R. Ng V. G. Wikander T. M. (2005). Peroxisome biogenesis disorders: the role of peroxisomes and metabolic dysfunction in developing brain. *J. Inherit. Metab. Dis.* 28 369–383. 10.1007/s10545-005-7059-y 15868469

[B18] Fujihara N. Sakaguchi A. Tanaka S. Fujii S. Tsuji G. Shiraishi T. (2010). Peroxisome biogenesis factor PEX13 is required for appressorium-mediated plant infection by the anthracnose fungus *Colletotrichum orbiculare*. *Mol. Plant Microbe Interact.* 23 436–445. 10.1094/MPMI-23-4-0436 20192831

[B19] Gale L. R. Ward T. J. Balmas V. Kistler H. C. (2007). Population subdivision of *Fusarium graminearum* sensu stricto in the upper midwestern united states. *Phytopathology* 97 1434–1439. 10.1094/PHYTO-97-11-1434 18943513

[B20] Gavric O. Santos D. B. D. Griffiths A. (2007). Mutation and divergence of the phospholipase C gene in *Neurospora crassa*. *Fungal Genet. Biol.* 44 242–249. 10.1016/j.fgb.2006.09.010 17157541

[B21] Goh J. Jeon J. Kim K. S. Park J. Park S. Y. Lee Y. H. (2011). The PEX7-mediated peroxisomal import system is required for fungal development and pathogenicity in *Magnaporthe oryzae*. *PLos One* 6:e28220. 10.1371/journal.pone.0028220 22194815 PMC3237427

[B22] Gould S. J. Mccollum D. Spong A. P. Heyman J. A. Subramani S. (2010). Development of the yeast *Pichia pastoris* as a model organism for a genetic and molecular analysis of peroxisome assembly. *Yeast* 8 613–628. 10.1002/yea.320080805 1441741

[B23] Heinemann S. Symoens F. Gordts B. Jannes H. Nolard N. (2004). Environmental investigations and molecular typing of *Aspergillus flavus* during an outbreak of postoperative infections. *J. Hosp. Infect.* 57 149–155. 10.1016/j.jhin.2004.02.007 15183246

[B24] Hiltunen J. K. Mursula A. M. Rottensteiner H. Wierenga R. K. Kastaniotis A. J. Gurvitz A. (2003). The biochemistry of peroxisomal β-oxidation in the yeast *Saccharomyces cerevisiae*. *FEMS Microbiol. Rev.* 27 35–64. 12697341 10.1016/S0168-6445(03)00017-2

[B25] Hou Z. Xue C. Peng Y. Katan T. Kistler H. C. Xu J. R. (2002). A mitogen-activated protein kinase gene (*MGV1*) in *Fusarium graminearum* is required for female fertility, heterokaryon formation, and plant infection. *Mol. Plant Microbe Interact.* 15 1119–1127. 10.1094/MPMI.2002.15.11.1119 12423017

[B26] Hu J. Baker A. Bartel B. Linka N. Mullen R. T. Reumann S. (2012). Plant peroxisomes: biogenesis and function. *Plant Cell* 24 2279–2303. 10.1105/tpc.112.096586 22669882 PMC3406917

[B27] Hynes M. J. Murray S. L. Khew G. S. Davis M. A. (2008). Genetic analysis of the role of peroxisomes in the utilization of acetate and fatty acids in *Aspergillus nidulans*. *Genetics* 178 1355–1369. 10.1534/genetics.107.085795 18245820 PMC2278063

[B28] Imazaki A. Tanaka A. Harimoto Y. Yamamoto M. Akimitsu K. Park P. (2010). Contribution of peroxisomes to secondary metabolism and pathogenicity in the fungal plant pathogen *Alternaria alternata*. *Eukaryot Cell* 9 682–694. 10.1128/EC.00369-09 20348386 PMC2863954

[B29] Jenczmionka N. J. Maier F. J. Lösch A. P. Schäfer W. (2003). Mating, conidiation and pathogenicity of *Fusarium graminearum*, the main causal agent of the head-blight disease of wheat, are regulated by the MAP kinase *gpmk1*. *Curr. Genet.* 43 87–95. 10.1007/s00294-003-0379-2 12695848

[B30] Jiang C. Zhang S. Zhang Q. Tao Y. Wang C. Xu J. R. (2015). FgSKN7 and FgATF1 have overlapping functions in ascosporogenesis, pathogenesis and stress responses in *Fusarium graminearum*. *Environ. Microbiol.* 17 1245–1260. 10.1111/1462-2920.12561 25040476

[B31] Kiel J. A. Veenhuis M. van der Klei I. J. (2006). PEX genes in fungal genomes: common, rare or redundant. *Traffic* 7 1291–1303. 10.1111/j.1600-0854.2006.00479.x 16978390

[B32] Koller A. Snyder W. B. Faber K. N. Wenzel T. J. Rangell L. Keller G. A. (1999). Pex22p of Pichia pastoris, essential for peroxisomal matrix protein import, anchors the ubiquitin-conjugating enzyme. Pex4p, on the peroxisomal membrane. *J. Cell Biol.* 146 99–112. 10.1083/jcb.146.1.99 10402463 PMC2199742

[B33] Kong X. Zhang H. Wang X. van der Lee T. Waalwijk C. van Diepeningen A. (2019). FgPex3, a peroxisome biogenesis factor, is involved in regulating vegetative growth, conidiation, sexual development, and virulence in *Fusarium graminearum*. *Front. Microbiol.* 10:2088. 10.3389/fmicb.2019.02088 31616386 PMC6764106

[B34] Kubo Y. Fujihara N. Harata K. Neumann U. Robin G. P. O’Connell R. (2015). *Colletotrichum orbiculare* FAM1 encodes a novel Woronin body-associated Pex22 peroxin required for appressorium-mediated plant infection. *mBio* 6 e1305–e1315. 10.1128/mBio.01305-15 26374121 PMC4600112

[B35] Law K. B. Bronte-Tinkew D. Di Pietro E. Snowden A. Jones R. O. Moser A. (2017). The peroxisomal AAA ATPase complex prevents pexophagy and development of peroxisome biogenesis disorders. *Autophagy* 13 868–884. 10.1080/15548627.2017.1291470 28521612 PMC5446072

[B36] Lazarow P. Fujiki Y. (1985). Biogenesis of peroxisomes. *Annu. Rev. Cell Biol.* 1 489–530. 3916321 10.1146/annurev.cb.01.110185.002421

[B37] Leslie J. F. Summerell B. A. (2007). *The Fusarium Laboratory Manual.* Hoboken, NJ: Wiley.

[B38] Li L. Wang J. Chen H. Chai R. Zhang Z. Mao X. (2017). Pex14/17, a filamentous fungus specific peroxin, is required for the import of peroxisomal matrix proteins and full virulence of *Magnaporthe oryzae*. *Mol. Plant Pathol.* 18 1238–1252. 10.1111/mpp.12487 27571711 PMC6638247

[B39] Li L. Wang J. Zhang Z. Wang Y. Liu M. Jiang H. (2014). MoPex19, which is essential for maintenance of peroxisomal structure and woronin bodies, is required for metabolism and development in the rice blast fungus. *PLos One* 9:e85252. 10.1371/journal.pone.0085252 24454828 PMC3891873

[B40] Liu F. F. Ng S. K. Lu Y. (2008). Making two organelles from one: woronin body biogenesis by peroxisomal protein sorting. *J. Cell Biol.* 180 325–339. 10.1083/jcb.200705049 18227279 PMC2213590

[B41] Livak K. J. Schmittgen T. D. (2001). Analysis of relative gene expression data using real-time quantitative PCR and the 2^–ΔΔ*CT*^ Method. *Methods* 25 402–408. 10.1006/meth.2001.1262 11846609

[B42] Lu J. P. Liu X. H. Feng X. X. Min H. Lin F. C. (2009). An autophagy gene, MgATG5, is required for cell differentiation and pathogenesis in Magnaporthe oryzae. *Curr. Genet.* 55 461–473. 10.1007/s00294-009-0259-5 19629489

[B43] Managadze D. Würtz C. Sichting M. Niehaus G. Veenhuis M. Rottensteiner H. (2010). The peroxin *PEX14* of *Neurospora crassa* is essential for the biogenesis of both glyoxysomes and Woronin bodies. *Traffic* 8 687–701. 10.1111/j.1600-0854.2007.00560.x 17461798

[B44] Matsuzono Y. Kinoshita N. Tamura S. Shimozawa N. Hamasaki M. Ghaedi K. (1999). Human PEX19: cDNA cloning by functional complementation, mutation analysis in a patient with Zellweger syndrome, and potential role in peroxisomal membrane assembly. *Proc. Natl. Acad. Sci. U.S.A.* 96 2116–2121. 10.1073/pnas.96.5.2116 10051604 PMC26746

[B45] Meinecke M. Cizmowski C. Schliebs W. Krüger V. Beck S. Wagner R. (2010). The peroxisomal importomer constitutes a large and highly dynamic pore. *Nat. Cell. Biol.* 12 273–277. 10.1038/ncb2027 20154681

[B46] Min K. Son H. Lee J. Choi G. J. Kim J. C. Lee Y. W. (2012). Peroxisome function is required for virulence and survival of *Fusarium graminearum*. *Mol. Plant Microbe Interact.* 25 1617–1627. 10.1094/MPMI-06-12-0149-R 22913493

[B47] Mirocha C. J. Kolaczkowski E. Xie W. Yu H. Jelen H. (1998). Analysis of deoxynivalenol and its derivatives (batch and single kernel) using gas chromatography/M\mass spectrometry. *J. Agric. Food Chem.* 46 1414–1418. 10.1021/jf970857o

[B48] Negoro H. Sakamoto M. Kotaka A. Matsumura K. Hata Y. (2018). Mutation in the peroxin-coding gene PEX22 contributing to high malate production in *Saccharomyces cerevisiae*. *J. Biosci. Bioeng.* 125 211–217. 10.1016/j.jbiosc.2017.08.010 28919252

[B49] Pestka J. J. Smolinski A. T. (2005). Deoxynivalenol: toxicology and potential effects on humans. *J. Toxicol. Environ. Health B* 8 39–69. 10.1080/10937400590889458 15762554

[B50] Pieuchot L. Jedd G. (2012). Peroxisome assembly and functional diversity in eukaryotic microorganisms. *Annu. Rev. Microbiol.* 66 237–263. 10.1146/annurev-micro-092611-150126 22994494

[B51] Platta H. W. Debelyy M. O. El Magraoui F. Erdmann R. (2008). The AAA peroxins Pex1p and Pex6p function as dislocases for the ubiquitinated peroxisomal import receptor Pex5p. *Biochem. Soc. Trans.* 36(Pt 1) 99–104. 10.1042/BST0360099 18208394

[B52] Platta H. W. El Magraoui F. Schlee D. Grunau S. Girzalsky W. Erdmann R. (2007). Ubiquitination of the peroxisomal import receptor Pex5p is required for its recycling. *J. Cell Biol.* 177 197–204. 10.1083/jcb.200611012 17452527 PMC2064128

[B53] Qin J. Wang G. Jiang C. Xu J. R. Wang C. (2015). Fgk3 glycogen synthase kinase is important for development, pathogenesis, and stress responses in *Fusarium graminearum*. *Sci. Rep.* 5:8504. 10.1038/srep08504 25703795 PMC4336942

[B54] Ramospamplona M. Naqvi N. I. (2006). Host invasion during rice-blast disease requires carnitine-dependent transport of peroxisomal acetyl-CoA. *Mol. Microbiol.* 61 61–75. 10.1111/j.1365-2958.2006.05194.x 16824095

[B55] Seong K. Hou Z. Tracy M. Kistler H. C. Xu J. R. (2005). Random insertional mutagenesis identifies genes associated with virulence in the wheat scab fungus *Fusarium graminearum*. *Phytopathology* 95 744–750. 10.1094/PHYTO-95-0744 18943005

[B56] Shabab M. (2013). Role of plant peroxisomes in protection against herbivores. *Subcell. Biochem.* 69 315–328. 10.1007/978-94-007-6889-5_17 23821156

[B57] Stanley W. A. Filipp F. V. Kursula P. Schüller N. Erdmann R. Schliebs W. (2006). Recognition of a functional peroxisome type 1 target by the dynamic import receptor pex5p. *Mol. Cell* 24 653–663. 10.1016/j.molcel.2006.10.024 17157249 PMC5030714

[B58] Suasteolmos F. Ziriónmartínez C. Takanorojas H. Perazareyes L. (2018). Meiotic development initiation in the fungus *Podospora anserina* requires the peroxisome receptor export machinery. *Biochim. Biophys. Acta Mol. Cell Res.* 1865 572–586. 10.1016/j.bbamcr.2018.01.003 29307785

[B59] Tenney K. Hunt I. Sweigard J. Pounder J. I. McClain C. Bowman E. J. (2000). Hex-1, a gene unique to filamentous fungi, encodes the major protein of the Woronin body and functions as a plug for septal pores. *Fungal Genet. Biol.* 31 205–217. 10.1006/fgbi.2000.1230 11273682

[B60] Wanders R. J. (2004). Peroxisomes, lipid metabolism, and peroxisomal disorders. *Mol. Genet. Metab.* 83 16–27. 10.1016/j.ymgme.2004.08.016 15464416

[B61] Wanders R. J. Waterham H. R. (2010). Peroxisomal disorders I: biochemistry and genetics of peroxisome biogenesis disorders. *Clin. Genet.* 67 107–133. 10.1111/j.1399-0004.2004.00329.x 15679822

[B62] Wang C. F. Zhang S. J. Hou R. Zhao Z. T. Zheng Q. Xu Q. J. (2011). Functional analysis of the kinome of the wheat scab fungus *Fusarium graminearum*. *PLoS Pathog.* 7:e1002460. 10.1371/journal.ppat.1002460 22216007 PMC3245316

[B63] Wang D. Visser N. V. Veenhuis M. van der Klei I. J. (2003). Physical interactions of the peroxisomal targeting signal 1 receptor pex5p, studied by fluorescence correlation spectroscopy. *J. Biol. Chem.* 278 43340–43345. 10.1074/jbc.M307789200 12930827

[B64] Wang J. Y. Li L. Chai R. Y. Qiu H. P. Zhang Z. Wang Y. L. (2019). Pex13 and Pex14, the key components of the peroxisomal docking complex, are required for peroxisome formation, host infection and pathogenicity-related morphogenesis in *Magnaporthe oryzae*. *Virulence* 10 292–314. 10.1080/21505594.2019.1598172 30905264 PMC6527019

[B65] Wang J. Li L. Zhang Z. Qiu H. Li D. Fang Y. (2015). One of three Pex11 family members is required for peroxisomal proliferation and full virulence of the rice blast fungus *Magnaporthe oryzae*. *PLoS One* 10:e0134249. 10.1371/journal.pone.0134249 26218097 PMC4517885

[B66] Wang J. Zhang Z. Wang Y. Li L. Chai R. Mao X. (2013). PTS1 peroxisomal import pathway plays shared and distinct roles to PTS2 pathway in development and pathogenicity of *Magnaporthe oryzae*. *PLoS One* 8:e55554. 10.1371/journal.pone.0055554 23405169 PMC3566003

[B67] Wang L. N. Zhang L. Liu C. J. Sun S. H. Liu A. X. Liang Y. C. (2020). The roles of FgPEX2 and FgPEX12 in virulence and lipid metabolism in *Fusarium graminearum*. *Fungal. Genet. Biol.* 135:103288. 10.1016/j.fgb.2019.103288 31704369

[B68] Wang Z. Y. Soanes D. M. Kershaw M. J. Talbot N. J. (2007). Functional analysis of lipid metabolism in *Magnaporthe grisea* reveals a requirement for peroxisomal fatty acid beta-oxidation during appressorium-mediated plant infection. *Mol. Plant Microbe Interact.* 20 475–491. 10.1094/MPMI-20-5-0475 17506326

[B69] Williams C. van den Berg M. Panjikar S. Stanley W. A. Distel B. Wilmanns M. (2012). Insights into ubiquitin-conjugating enzyme co-activator interactions from the structure of the Pex4p:Pex22p complex. *EMBO J.* 31 391–402. 10.1038/emboj.2011.411 22085930 PMC3261564

[B70] Zhang F. Geng L. P. Huang L. H. Deng J. L. Opemipo E. F. Guangshan Y. (2018). Contribution of peroxisomal protein importer AflPex5 to development and pathogenesis in the fungus *Aspergillus flavus*. *Curr. Genet.* 64 1335–1348. 10.1007/s00294-018-0851-7 29869688

[B71] Zhang L. Liu C. Wang L. Sun S. Liu A. Liang Y. (2019a). FgPEX1 and FgPEX10 are required for the maintenance of Woronin bodies and full virulence of *Fusarium graminearum*. *Curr. Genet.* 65 1383–1396. 10.1007/s00294-019-00994-8 31111312

[B72] Zhang L. Wang L. Liang Y. Yu J. (2019b). FgPEX4 is involved in development, pathogenicity, and cell wall integrity in *Fusarium graminearum*. *Curr. Genet.* 65 747–758. 10.1007/s00294-018-0925-6 30603875

[B73] Zhao X. Tang B. Xu J. Wang N. Zhou Z. Zhang J. (2020). A SET domain-containing protein involved in cell wall integrity signaling and peroxisome biogenesis is essential for appressorium formation and pathogenicity of *Colletotrichum gloeosporioides*. *Fungal Genet. Biol.* 29:103474. 10.1016/j.fgb.2020.103474 33007450

[B74] Zhou X. Li G. Xu J. R. (2011). Efficient approaches for generating GFP fusion and epitope-tagging constructs in filamentous fungi. *Methods Mol. Biol.* 722 199–212. 10.1007/978-1-61779-040-9_15 21590423

[B75] Zolman B. K. Monroe-Augustus M. Silva I. D. Bartel B. (2005). Identification and functional characterization of *Arabidopsis* PEROXIN4 and the interacting protein PEROXIN22. *Plant Cell* 17 3422–3435. 10.1105/tpc.105.035691 16272432 PMC1315379

