# Supplementary material for: Peroxin FgPEX22-Like Is Involved in FgPEX4 Tethering and Fusarium graminearum Pathogenicity
Source: Front Microbiol. 2021 Dec 10;12:756292. doi: 10.3389/fmicb.2021.756292 (PMC8702864; doi:10.3389/fmicb.2021.756292)
Supplement: Supplementary file 1 [file Table_1.DOCX]

Supplementary Material

## Supplementary Figures


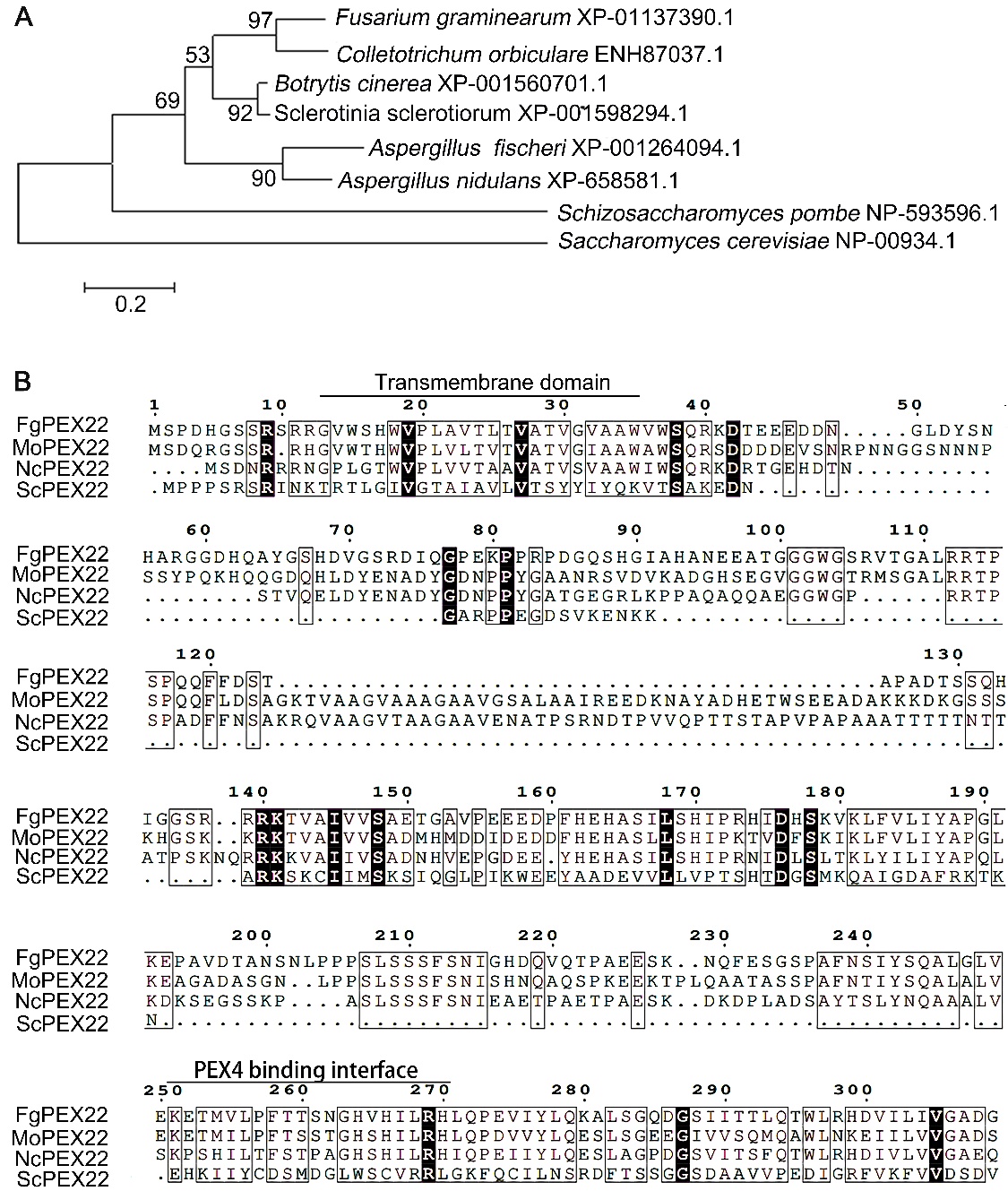


**Supplementary Figure 1.** Phylogenetic analysis of PEX22-like in *F. graminearum* and other fungi. (A) phylogenetic tree was generated based on the neighbor-joining method using MEGA version 6.0. The numbers at nodes represent the percentage of their occurrence in 1000 bootstrap replicates. The accession numbers are shown after the species names. (B) Alignment of PEX22 amino acid sequences of *Fusarium graminearum* (FgPEX22-like), *Magnaporthe oryzae* (MoPEX22), *Neurospora crassa* (NcPEX22), and *Saccharomyces cerevisiae* (ScPEX22). Line denotes the Transmembrane domain and PEX4 binding interface.


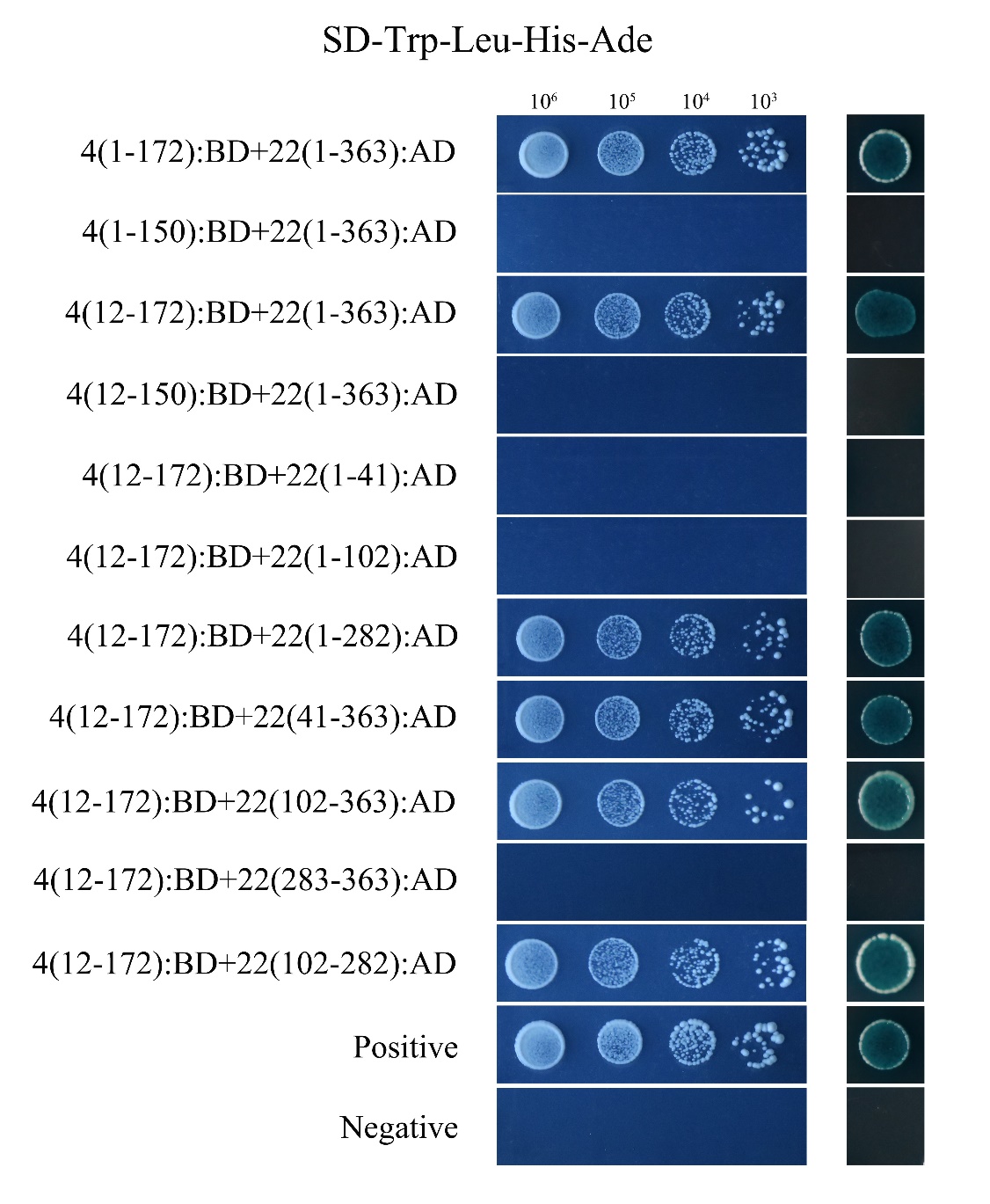


**Supplementary Figure 2.** The interaction of FgPEX4 and FgPEX22-like in key action regions. The truncated versions of FgPEX22-like and pGADT7 were created as prey vectors named FgPEX22-like (a–b)-ADs. The truncated versions of FgPEX4 and pGBKT7 were constructed as bait vectors named FgPEX4 (c–d)-BDs. Yeast two-hybrid assays were performed to detect the interactions between FgPEX22-like (a–b)-ADs and FgPEX4 (c–d)-BDs with various combinations. The results indicate the minimal region required for FgPEX4 binding consists of residues 102–282. The first 11 amino acids of FgPex4 were not required for FgPEX22-like binding. 22 and 4 in figure referring to FgPEX22-like and FgPEX4 respectively.


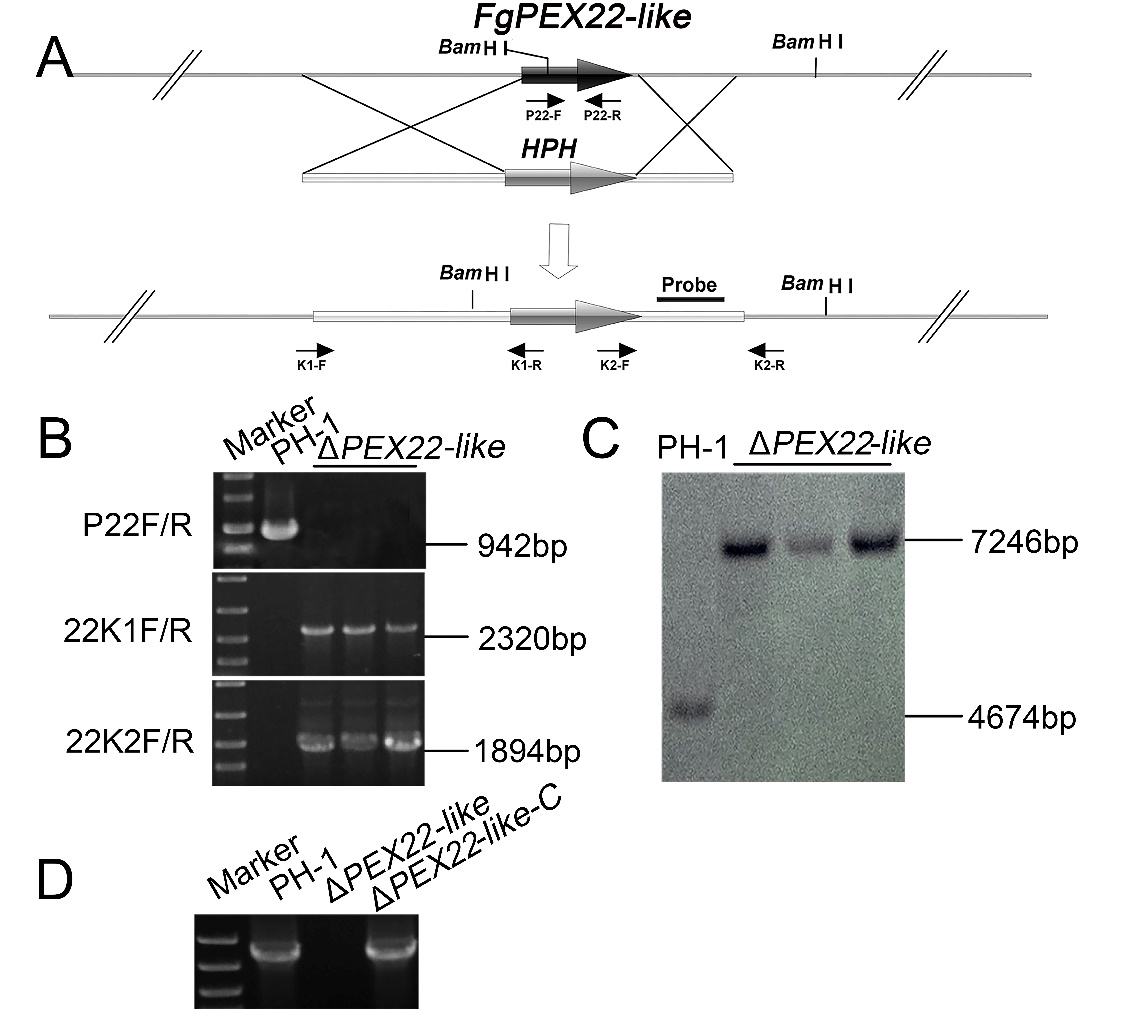


**Supplementary Figure 3.** The *FgPEX22-like* gene deletion strategy and confirmation in *F. graminearum.* (A) Schematic representation of the homologous recombination strategy used for mutant construction. The ∆*PEX22-like* mutants were created by replacing the *FgPEX22-like* genes with hygromycin phosphotransferase cassette (*HPH*). The specific restriction enzyme *Bam*H I were marked on the downstream flanking. The deletion cassettes including the respective upstream and downstream flanking sequences together with the *hph* cassette were amplified and transformed into the wild-type strain PH-1. The putative mutants were screened with diagnostic primers, which were indicated with arrows. (B) PCR analyses showed that the ∆*PEX22-like* mutants were correctly constructed. (C) Southern blot analysis of ∆*PEX22-like*. Genomic DNA was digested with *EcoR* I. The downstream sequences were used as the probe for Southern blot analysis. (D) PCR analysis of the targeted *FgPEX22-like* gene with P22F/R primers in *FgPEX22-like* *-*complementation strain.


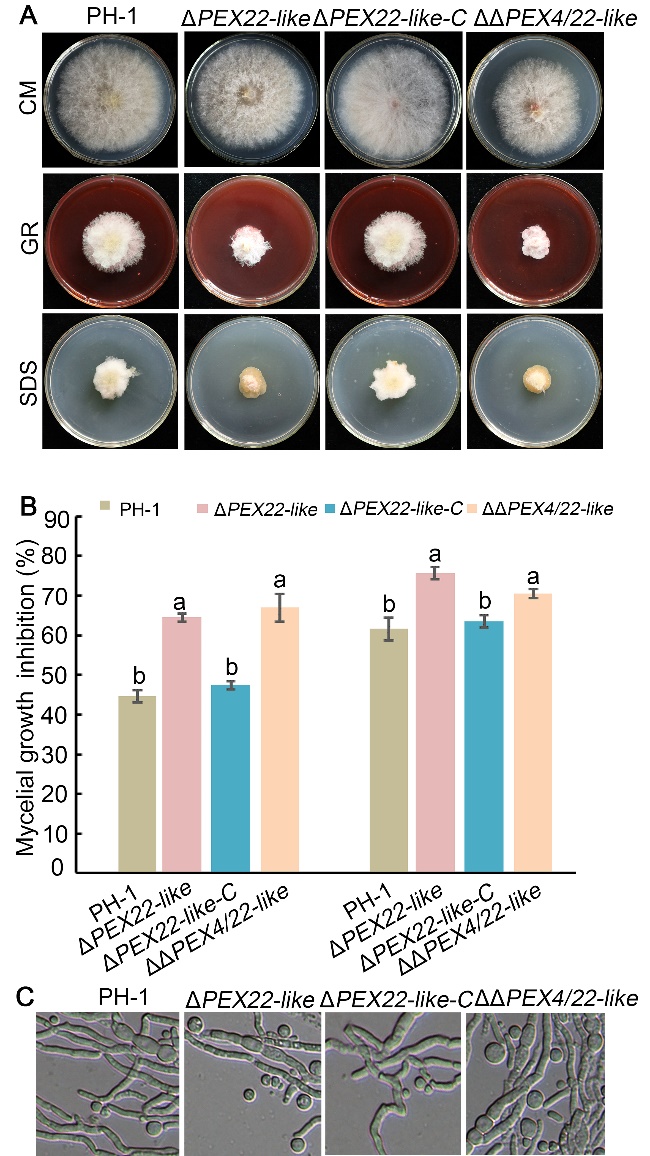


**Supplementary Figure 4.** Tolerance test of *F. graminearum* mutants *∆PEX22-like* and *∆∆PEX4/22-like* to cell wall and cell membrane interference agents. (**a**) *F. graminearum* strain PH-1, ∆*PEX22-like*, ∆*PEX22-like -*C, and *∆∆PEX4/22-like* strains were grown on complete medium (CM) containing 0.2 μg/L Congo red (CR) or 0.01% SD for 3 d at 25°C. (**b**) Percent inhibition of PH-1, ∆*PEX22-like*, ∆*PEX22-like -*C, and *∆∆PEX4/22-like* were measured. % Inhibition =(D-d)/D × 100%, where D=diameter of untreated strain and d=diameter of treated strain. **(c)** Mycelia of all strains treated with driselase and lysozyme for 30 min at 30°C. Cell walls of ∆*PEX22-like* and *∆∆PEX4/22-like* were severely damaged and the digested mycelia released large amounts of protoplasts. Bar = 20 μm.


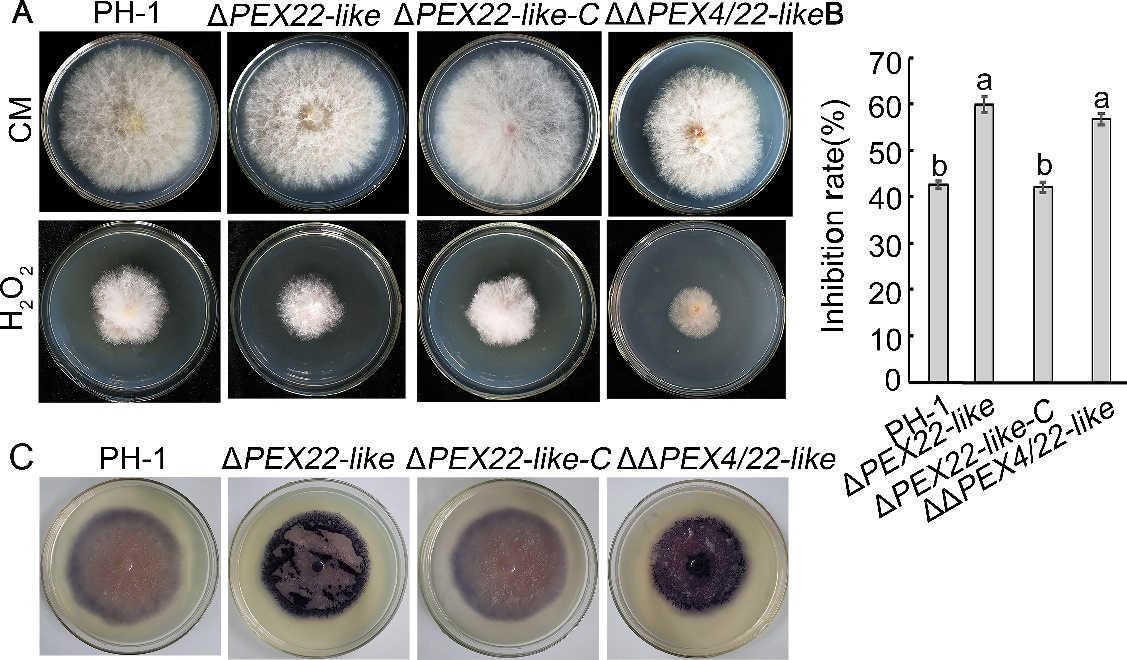


**Supplementary Figure 5.** Defects in ROS elimination in *F. graminearum* mutants ∆*PEX22-like* and *∆∆PEX4/22-like*. (A) Mycelial growth of *F. graminearum* strain PH-1, ∆*PEX22-like*, ∆*PEX22-like-*C, and *∆∆PEX4/22-like* were cultured on complete medium (CM) containing 20 mM H_2_O_2_ for 3 d. (B) Mycelial growth inhibition was measured and statistically analyzed. Line bars in each column denote standard deviations of three repeated experiments. (C) Nitroblue tetrazolium (NBT) staining for ROS production in mycelia of PH-1, ∆*PEX22-like*, ∆*PEX22-like-*C, and *∆∆PEX4/22-like* strains. The dark color of the colony indicated increased ROS production.

## Supplementary Tables

**Supplementary Table 1.** Primers used in this study.

| Primer Sequence (5′-3′) Application | | |
| --- | --- | --- |
| P4-AD-F | GGAGGCCAGTGAATTATGCCACCTTCGTCATCA | Vector construction of PEX4-pGADT7AD  （549bp） |
| P4-AD-R | CGAGCTCGATGGATCTGGTCCCTCGTATCTCGA |  |
| P4-BD-F | CATGGAGGCCGAATTATGCCACCTTCGTCATCA | Vector construction of PEX4- pGBKT7 BD  （549bp） |
| P4-BD-R | TAGTTATGCGGCCGCTGGTCCCTCGTATCTCGA |  |
| P22-AD-F | GGAGGCCAGTGAATTATGTCGCCCGATCATGGATC | Vector construction of PEX22-pGADT7 AD  (1122bp) |
| P22-AD-R | CGAGCTCGATGGATCCTCACGTCCCTGAACTCGAC |  |
| P22-BD-F | CATGGAGGCCGAATTATGTCGCCCGATCATGGATC | Vector construction of PEX22- pGBKT7 BD(1122bp) |
| P22-BD-R | TAGTTATGCGGCCGCCTCACGTCCCTGAACTCGAC |  |
| P4(1-150)-BD-F | ATGGCCATGGAGGCCGAATTCATGCCACCTTCGTCATCAAACA | Vector construction of PEX4(1-150)-pGBKT7 BD (453bp) |
| P4(1-150)-BD-R | CTAGTTATGCGGCCGCTGCAGTCAATCACCCGAGCGCAG |  |
| P4(12-172)-BD-F | ATGGCCATGGAGGCCGAATTCATGTCGCGAAGTGCGAGTAAA | Vector construction of PEX4(12-172)-pGBKT7 BD (489bp) |
| P4(12-172)-BD-R | CTAGTTATGCGGCCGCTGCAGTCATGGTCCCTCGTATCTCGA |  |
| P4(12-150)-BD-F | ATGGCCATGGAGGCCGAATTCATGTCGCGAAGTGCGAGTAAA | Vector construction of PEX4(12-150)-pGBKT7 BD (423bp) |
| P4(12-150)-BD-R | CTAGTTATGCGGCCGCTGCAGTCAATCACCCGAGCGCAG |  |
| P22(1-41)-AD-F | GCCATGGAGGCCAGTGAATTCATGTCGCCCGATCATGGAT | Vector construction of PEX22(1-41)-pGADT7 AD (126bp) |
| P22(1-41)-AD-R | CAGCTCGAGCTCGATGGATCCTTACTTTCTCTGGCTCCATACCCA |  |
| P22(1-102)-AD-F | GCCATGGAGGCCAGTGAATTCATGTCGCCCGATCATGGAT | Vector construction of PEX22(1-102)-pGADT7 AD (309bp) |
| P22(1-102)-AD-R | CAGCTCGAGCTCGATGGATCCTTAGCCCCCGCCGGTTGC |  |
| P22(1-282)-AD-F | GCCATGGAGGCCAGTGAATTCATGTCGCCCGATCATGGAT | Vector construction of PEX22(1-282)-pGADT7 AD (849bp) |
| P22(1-282)-AD-R | CAGCTCGAGCTCGATGGATCCTTAAAGGGCTTTCTGAAGATAGATAACT |  |
| P22(41-363)-AD-F | GCCATGGAGGCCAGTGAATTCATGAAGGACACGGAAGAGGAGG | Vector construction of PEX22(41-363)- pGADT7 AD (975bp) |
| P22(41-363)-AD-R | CAGCTCGAGCTCGATGGATCCTTACTCACGTCCCTGAACTCGAC |  |
| P22(102-363)-AD-F | GCCATGGAGGCCAGTGAATTC ATGGGCTGGGGTTCGCGC | Vector construction of PEX22(102-363)-pGADT7 AD (792bp) |
| P22(102-363)-AD-R | CAGCTCGAGCTCGATGGATCC TTACTCACGTCCCTGAACTCGAC |  |
| P22(283-363)-AD-F | GCCATGGAGGCCAGTGAATTC ATGTCTGGTCAAGATGGCAGCA | Vector construction of PEX22(283-363)-pGADT7 AD (249bp) |
| P22(283-363)-AD-R | CAGCTCGAGCTCGATGGATCC TTACTCACGTCCCTGAACTCGAC |  |
| 4-GFP-F | TCACTATAGGGCGAATTGGGTACTCAAATTGGGGCAGAGGAGAACAGAAGG | Vector construction of  PEX4 – pFL2 |
| 4-GFP-R | CCACCCCGGTGAACAGCTCCTCGCCCTTGCTTTGCTGAAGTTGAAGGCTTC |  |
| 22-Flag-F | CGACTCACTATAGGGCGAATTGGGTACTCAAATTGTTTCCATTCGCTCGTTGTCC | Vector onstruction of PEX22- pFL7 |
| 22-Flag-R | CTTTATAATCACCGTCATGGTCTTTGTAGTCCTCACGTCCCTGAACTCG |  |
| PFL2 insert-F | TAACGCCAGGGTTTTCCCAGTCA | Verification of  PEX4 – pFL2 |
| PFL2 insert-R | GTCTTGACTGGCTCGTGAATG |  |
| PFL7 insert-F | TAGTAACCTTCCGCCACCTTCG | Verification of  PEX22- pFL7 |
| PFL7 insert-R | GTCATCGTCATCCTTGTAATCGA |  |
| HYG-F | GGCTTGGCTGGAGCTAGTGGAGGTCAA | the front of *hph* fragment (764 bp) |
| HY-R | GTATTGACCGATTCCTTGCGGTCCGAA |  |
| YG-F | GATGTAGGAGGGCGTGGATATGTCCT | the later of *hph* fragment (930 bp) |
| HYG-R | AACCCGCGGTCGGCATCTACTCTATTC |  |
| HYG-R | AACCCGCGGTCGGCATCTACTCTATTC |  |
| pex22-AF | AACCAAGTCGCAAGGCAGAA | 5’-flanking of FgPEX-22like gene(931bp) |
| pex22-AR | TTGACCTCCACTAGCTCCAGCCAAGCCTAATCAGCGTCAATAACACAG |  |
| pex22-BF | GAAATGAGTAGATGCCGACCGCGGGTTAACACGAGCACGCGTGTGTAC | 3’-flanking of FgPEX22 gene(671bp) |
| pex22-BR | TGGCGGACATTGATGGTGA |  |
| pex22-YF | TAGCCGAACACTAAGGGAC | FgPEX 22 like fragment (942bp) |
| pex22-YR | TTGGACATTTCACCAGGAT |  |
| pex22-K1F | TTTCCATTCGCTCGTTGTCC | Identification of FgPEX22 deletion transformant (2320 bp) |
| pex22-K1R | GTCCTCGTTCCTGTCTGCTAAT |  |
| pex22-K2F | CACTGGCAAACTGTGATGG | Identification of FgPEX22 deletion transformant (1894 bp) |
| pex22-K2R | CTGCGATGCTGTTTCTCGTC |  |
| pex22-TZF | TGACAAACCCAACTCCATT | probe in Southern blot analysis (348 bp) |
| pex22-TZR | GACCATACGACTGACCACC |  |
| 4GFP-F  4GFP-R | ACTCACTATAGGGCGAATTGGGTACTCAAATTGGTTGGCAGAGGAGAACAGAAGG  CACACCCCGGTGAACAGCTCCTCGCCCTTGCTCACTGGTCCCTCGTATCTCGAATC | Subcellular localization of PEX4 |
| 22CF  22CR  PMP70-GFP-F  PMP70-GFP-R | AAGCTGGGCGTGTTGTCTGGGC  TGGCGGACATTGATGGTGACGG  ACTCACTATAGGGCGAATTGGG  TACTCAAATTGGTTAAGAAGACCCGACCGGAAGA  CACCACCCCGGTGAACAGCTCCTCGCCCTTGCTCACAGCGGCTGTTAATTCAGC | Complementation of  FgPEX22（3804 bp）  Subcellular localization of PMP70 (3841 bp)  Subcellular localization of HEX1 (4539 bp) |
| HEX1-GFP-F | ACTCACTATAGGGCGAATTGGGTACTCAAATTGGTTAGCTAATAATGGCAAGGAG |  |
| HEX1-GFP-R | CACCACCCCGGTGAACAGCTCCTCGCCCTTGCTCACGTTAAGTAGATGCGGAATC |  |
| G1F | GAGGTTGCGATTTCTCTGCCGTATCTG | the front of G418 fragment(764bp) |
| G1R | CCACAGTCGATGAATCCAGAAAAGCG |  |
| G2F | GGAAGGGACTGGCTGCTATTGG | the later of G418 fragment(930bp) |
| G2R | GCCAGCAGTAGACACTTGGAATCTAAAC |  |
| pex22-2-AF | AACCAAGTCGCAAGGCAGAA | 5’-flanking of FgPEX 22like gene(931bp) |
| pex22-2-AR | CAGATACGGCAGAGAAATCGCAACCTCTAATCAGCGTCAATAACACA |  |
| pex22-2-BF | GTTTAGATTCCAAGTGTCTACTGCTGGCAACACGAGCACGCGTGTGTAC | 3’-flanking of FgPEX22 gene(863bp) |
| pex22-2-BR | TGGCGGACATTGATGGTGA |  |
| TRI5-F | GAGTGTTTCATGCATGGCTACGTC | qRT-PCR primers of  *FgTRI5* |
| TRI5-R | CTGAGCCTCCTTCACATCGTCC |  |
| TRI6-F | CTGAGGGCATTCTGAGTAGCGACA | qRT-PCR primers of  *FgTRI6* |
| TRI6-R | CGTTATGTTTATCGGCACTTTG |  |
| TRI10-F | GCGACAGGAGCAAGAACATAA | qRT-PCR primers of  *FgTRI10* |
| TRI10-R | GGCGGCGTAAATCTGAGTG |  |

**Supplementary Table 2**  Summary of yeast tow-hybrid results.

| FgPEX22(a-b)-ADs | FgPEX4(c-d)-BDs | Interaction |
| --- | --- | --- |
| P22(1-363) (Full length) | P4(1-172) (Full length) | + |
| P22(1-363) | P4(1-150) | - |
| P22(1-363) | P4(12-172) | + |
| P22(1-363) | P4(12-150) | - |
| P22(1-41) | P4(12-172) | - |
| P22(1-102) | P4(12-172) | - |
| P22(1-282) | P4(12-172) | + |
| P22(41-363) | P4(12-172) | + |
| P22(102-363) | P4(12-172) |  |
| P22(282-363) | P4(12-172) | - |
